# Supplementary material for: Structural basis of CSN-mediated SCF deneddylation
Source: Nat Commun. 2026 Jan 23;17:951. doi: 10.1038/s41467-025-67566-y (PMC12848000; doi:10.1038/s41467-025-67566-y)
Supplement: Supplementary file 1 — Supplementary information [file 41467_2025_67566_MOESM1_ESM.pdf]

Table of Contents

SUPPLEMENTARY FIGURES 4

SUPPLEMENTARY FIG. 1. RECONSTITUTION, CRYO-ELECTRON MICROSCOPY, AND SINGLE PARTICLE ANALYSIS OF CSN^{5H138A}-^{N8}SCF. 5

SUPPLEMENTARY FIG. 2. FOCUSED 3D CLASSIFICATION OF THE SUBSTRATE RECEPTOR REGION IN THE CSN^{5H138A}-^{N8}SCF DATASET. 7

SUPPLEMENTARY FIG. 3. MODELS-TO-MAP FOURIER SHELL CORRELATION (FSC) ANALYSIS FOR THE CSN^{5H138A}-^{N8}SCF COMPLEXES 8

SUPPLEMENTARY FIG. 4. CSNAP IS AN INTEGRAL SUBUNIT OF THE CSN COMPLEX. 9

SUPPLEMENTARY FIG. 5. COMPARATIVE SPR ANALYSIS OF CSN VARIANTS BINDING TO NEDDYLATED AND NON-NEDDYLATED CUL1/RBX1 11

SUPPLEMENTARY FIG. 6. CONFORMATIONAL CHANGES IN CSN BETWEEN THE CSN^{APO} AND PRE-ACTIVATED CSN^{5H138A} STATES. 12

SUPPLEMENTARY FIG. 7. CONFORMATIONAL CHANGES IN ^{N8}SCF BETWEEN AN ACTIVE ^{N8}SCF UBIQUITIN-BOUND STATE AND THE PRE-ACTIVATED CSN^{5H138A} COMPLEX. 13

SUPPLEMENTARY FIG. 8. $CSN2^{ARM}$ AND $CSN4^{ARM}$ CLAMP $CUL1^{CTD}$ AND STABILISE $RBX1^{RING}$ IN PRE-ACTIVATED CSN^{5H138A}-^{N8}SCF. 14

SUPPLEMENTARY FIG. 9. STRUCTURAL COMPARISON OF PRE-ACTIVATED AND ACTIVATED CSN^{5H138A}-^{N8}SCF AT THE $RBX1^{RING}$ DOMAIN 15

SUPPLEMENTARY FIG. 10. CSN DIRECTLY COMPETES WITH KEY COMPONENTS OF THE CRL REGULATORY CYCLE. 16

SUPPLEMENTARY FIG. 11. SDS-PAGE (4-12%) ANALYSIS AND COOMASSIE STAINING OF PURIFIED MUTANTS. 17

Supplementary Fig. 12. SPR analysis of binding affinities between CUL1/RBX1 and CSN mutants. 22

Supplementary Fig. 13. *In vitro* deneddylation activity assays. 25

Supplementary Fig. 14. SPR kinetic analysis of CSN variants binding to neddylated CUL1/RBX1 variants 26

Supplementary Fig. 15. Conservation of key interaction residues in CUL1 and RBX1. 29

Supplementary Fig. 16. Structural analysis of SR recognition by CSN. 30

Supplementary Fig. 17. Structural docking of diverse SRs onto pre-activated CSN^{5H138A}-^{N8}SCF and CSN^{E104A}-SCF dissociation-state-3. 32

Supplementary Fig. 18. Structural versatility of $CSN5^{Ins-1}$. 33

Supplementary Fig. 19. Structural comparison of active sites in activated CSN^{5H138A}-^{N8}SCF and MPN^{+} metalloprotease family members. 34

Supplementary Fig. 20. Conservation analysis of R717 and R741 across the Cullin family. 35

Supplementary Fig. 21. CSN5 exhibits strict selectivity for NEDD8. 36

Supplementary Fig. 22. Cryo-EM and single particle analysis on CSN^{5E104A}-SCF dissociation states. 40

Supplementary Fig. 23. Models-to-map Fourier Shell Correlation (FSC) analysis for the CSN^{5E104A}-SCF dissociation states. 41

Supplementary Fig. 24. Cryo-EM structures from CSN^{E104A}-SCF dissociation states. 42

Supplementary Fig. 25. Structural comparison between activated CSN^{5H138A}-^{N8}SCF and CSN^{E104A}-SCF dissociation-state-2. 43

Supplementary Fig. 26. Visualisation of $RBX1^{RING}$ in unprocessed cryo-EM maps of CSN^{E104A}-SCF dissociation-state-2 and dissociation-state-4. 44

SUPPLEMENTARY FIG. 27. STRUCTURAL COMPARISON BETWEEN CSN^{E104A}-SCF DISSOCIATION-STATE-2 AND DISSOCIATION-STATE-3. 44

SUPPLEMENTARY FIG. 28. KEY INTERFACES DESCRIBING CSN^{E104A}-SCF DISSOCIATION-STATE-3. 45

SUPPLEMENTARY FIG. 29. CONFORMATIONAL CHANGES BETWEEN ACTIVATED CSN^{5H138A}-^{N8}SCF AND CSN^{E104A}-SCF DISSOCIATION-STATE-3. 45

SUPPLEMENTARY FIG. 30. STEPWISE CONFORMATIONAL CHANGES IN SCF, $CSN2^{ARM}$ AND $CSN4^{ARM}$ DURING CSN^{E104A}-SCF DISSOCIATION 46

SUPPLEMENTARY FIG. 31. CSN2-BOUND IP6 AND ITS INTERACTIONS WITH CUL1/RBX1 IN CSN^{E104A}-SCF DISSOCIATION-STATE-3. 47

SUPPLEMENTARY FIG. 32. STRUCTURAL COMPARISON BETWEEN CSN^{E104A}-SCF DISSOCIATION-STATE-3 AND DISSOCIATION-STATE-4. 47

SUPPLEMENTARY FIG. 33. STRUCTURAL REARRANGEMENTS IN CSN UPON CSNAP INCORPORATION 48

SUPPLEMENTARY TABLES 49

SUPPLEMENTARY TABLE 1 CRYO-EM DATA COLLECTION AND REFINEMENT STATISTICS OF CSN^{5H138A}-^{N8}SCF 49

SUPPLEMENTARY TABLE 2 CRYO-EM DATA COLLECTION AND REFINEMENT STATISTICS OF CSN^{5E104A}-SCF 51

SUPPLEMENTARY TABLE 3 OLIGONUCLEOTIDE SEQUENCES 53

UNCROPPED SDS-PAGE GELS IN SUPPLEMENTARY FIGURES 54

SUPPLEMENTARY REFERENCE 55

Supplementary Figures

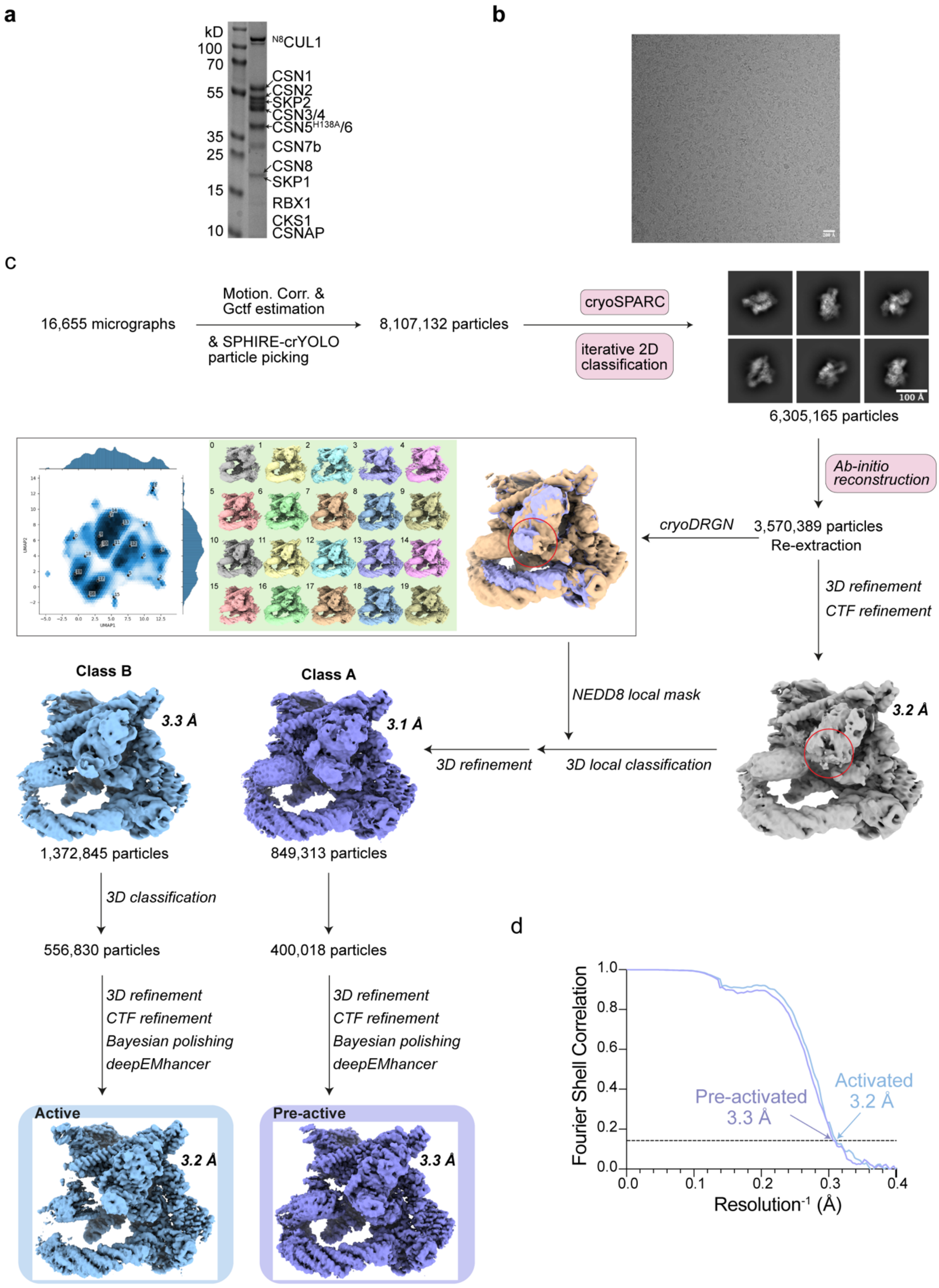

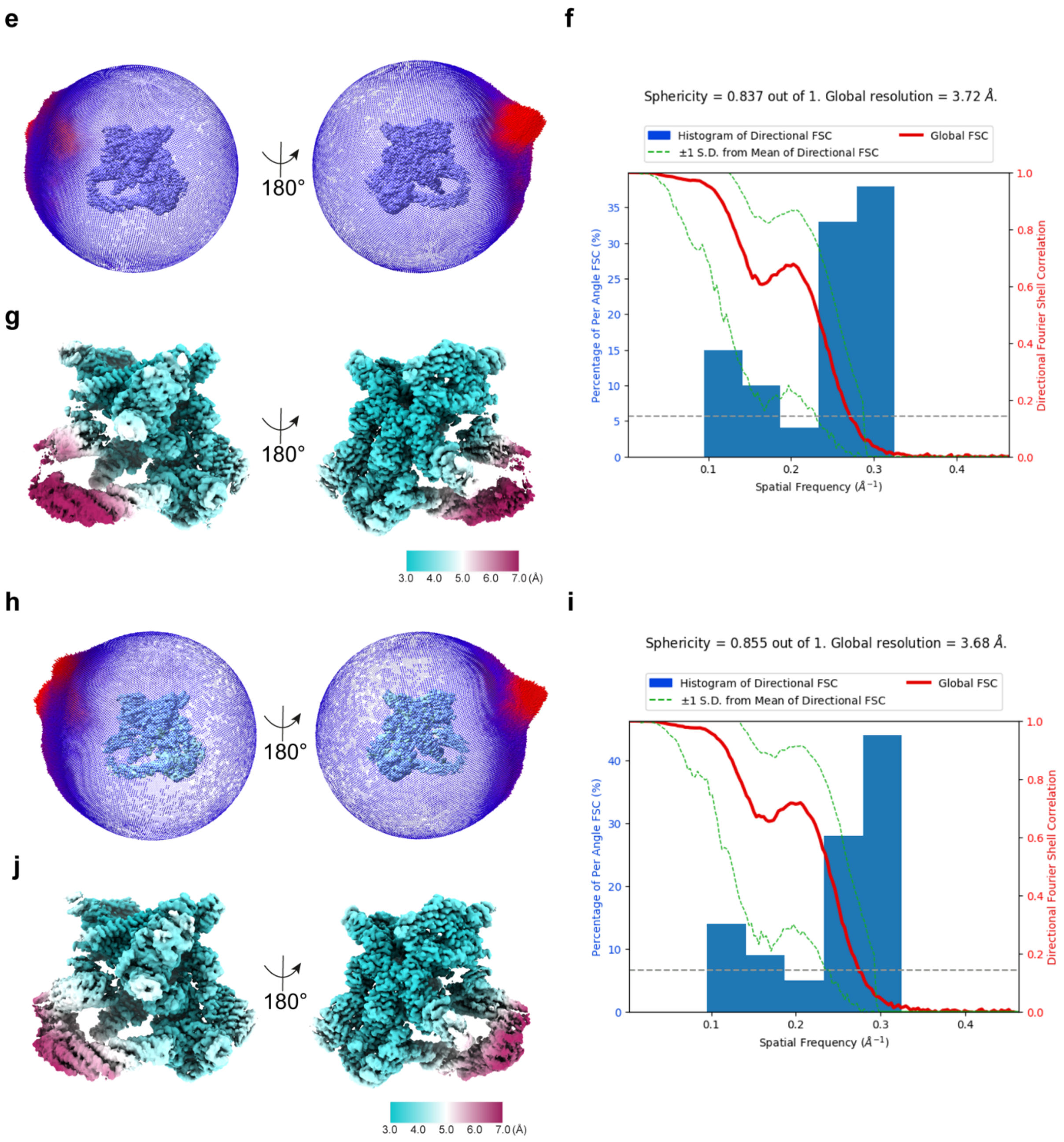

Supplementary Fig. 1. Reconstitution, cryo-electron microscopy, and single particle analysis of CSN^{5H138A}-^{N8}SCF.

a, Coomassie-stained SDS PAGE of the purified CSN^{5H138A}-^{N8}SCF protein complex. **b,** A representative motion-corrected micrograph and particle picking (red circles). **c,** Single particle analysis workflow for the CSN-^{N8}SCF dataset, resulting in 3D reconstructions of pre- and activated CSN^{5H138A}-^{N8}SCF. CryoDRGN-based heterogeneity analysis is shown in the inset. Left: UMAP projection of the latent space. Middle: Representative cryoDRGN-generated structures at indicated UMAP coordinates. Right: Overlay of structures 3 and 8 highlighting distinct NEDD8

positions (circled in red). Densities from structures 3 and 8 were combined to create a mask for 3D local classification. 3D local classification was performed in RELION-4.0 [1] utilising a NEDD8-specific mask (generated from classification results from CryoDRGN [2]). **d,** Resolution estimates of pre-activated and activated CSN^{5H138A}-^{N8}SCF. **e,** Euler angle distribution plots of pre-activated CSN^{5H138A}-^{N8}SCF complex. **f,** Directional 3DFSC plots of pre-activated CSN^{5H138A}-^{N8}SCF complex [3]. **g,** Local resolution estimates of pre-activated CSN^{5H138A}-^{N8}SCF complex. **h,** Euler angle distribution plots of activated CSN^{5H138A}-^{N8}SCF complex. **i,** Directional 3DFSC plots of activated CSN^{5H138A}-^{N8}SCF complex [3]. **j,** Local resolution estimates of activated CSN^{5H138A}-^{N8}SCF complex.

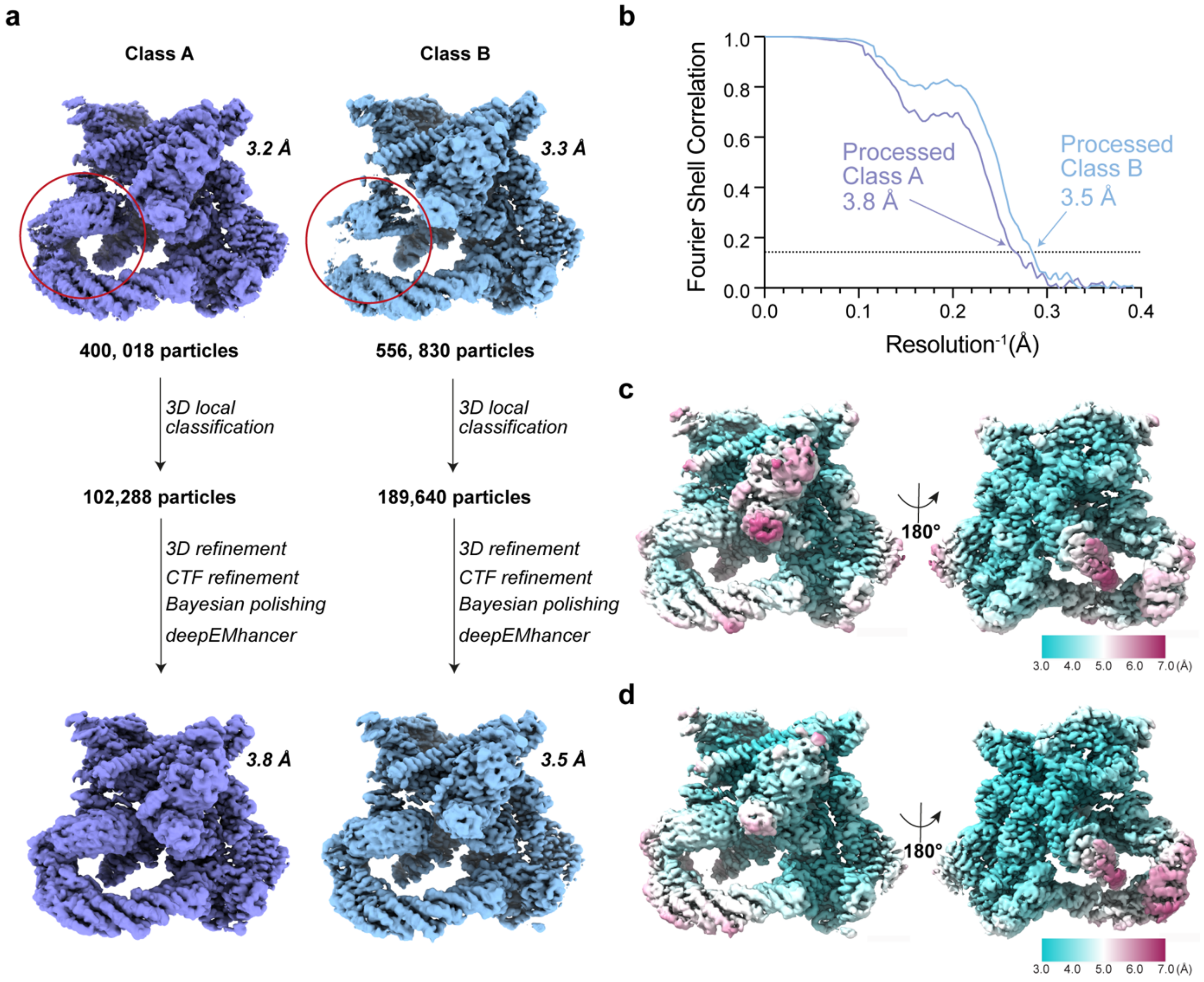

Supplementary Fig. 2. Focused 3D classification of the substrate receptor region in the CSN^{5H138A}-^{N8}SCF dataset.

a, Workflow of 3D local classification targeting the substrate receptor (SKP1–SKP2–CKS1) in both Class A and Class B sub-datasets. The red circle highlights the mask used for focused classification. **b,** Resolution estimates of the final maps in (**a**). **c-d,** Local resolution estimates of the map generated from Class A and Class B, respectively.

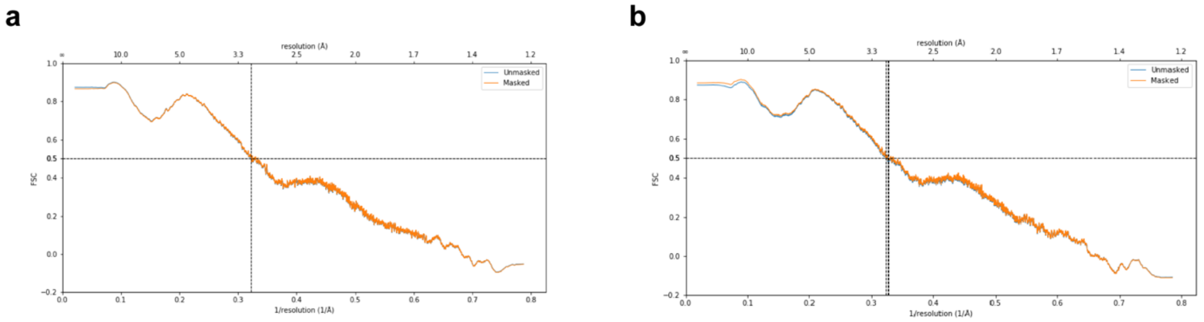

Supplementary Fig. 3. Models-to-map Fourier Shell Correlation (FSC) analysis for the CSN^{5H138A}-^{N8}SCF complexes.

a, FSC curves for the pre-activated CSN^{5H138A}–^{N8}SCF complex. **b**, FSC curves for the activated CSN^{5H138A}–^{N8}SCF complex. For each structure, the FSC was calculated between the refined atomic model and the corresponding cryo-EM map with Phenix version 1.21.1 [4]. Both masked (orange) and unmasked (blue) correlations are shown. The resolution at the FSC = 0.5 criterion is indicated by the vertical dashed line. The close agreement between masked and unmasked curves suggests good model-to-map correlation and minimal overfitting.

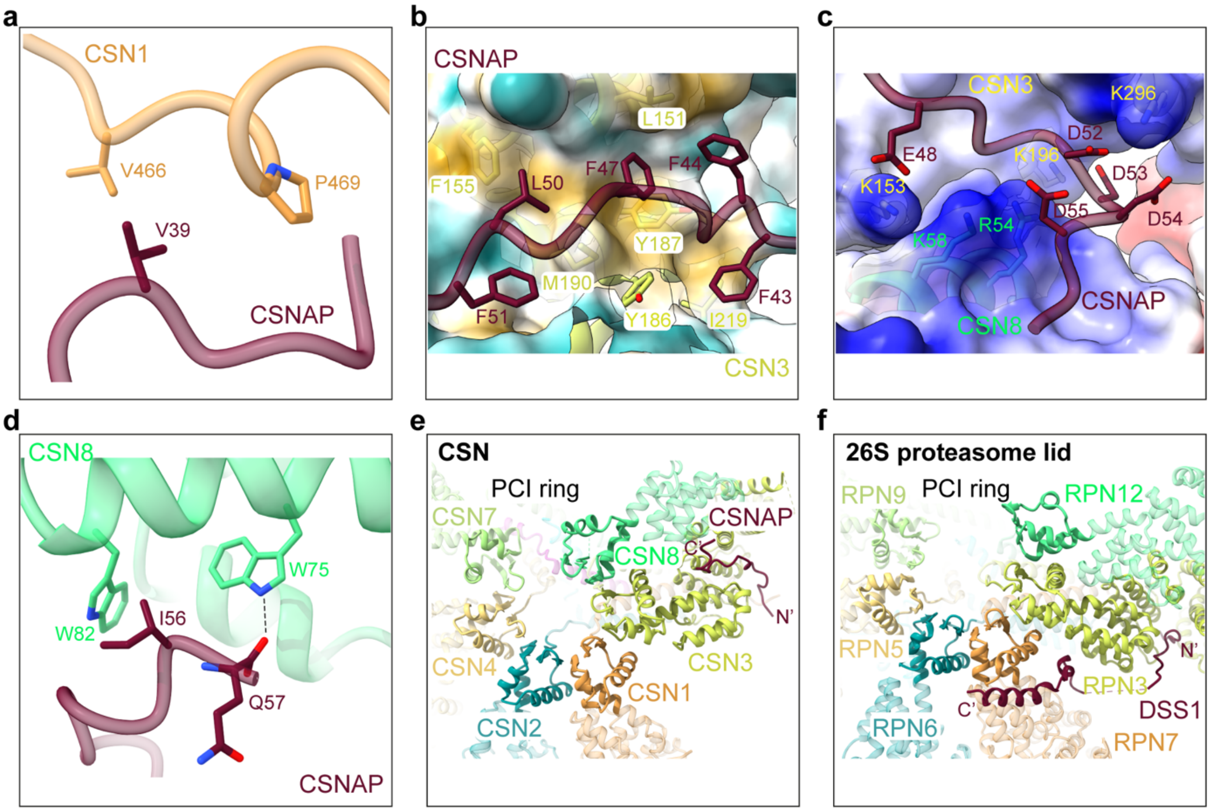

Supplementary Fig. 4. CSNAP is an integral subunit of the CSN complex.

a, Structural interactions between CSN1 and CSNAP. **b,** CSN3 engages CSNAP through hydrophobic interactions. CSN3 is displayed as a hydrophobic surface, with hydrophobicity mapped using a colour scale (yellow denoting hydrophobic residues and cyan for hydrophilic residues). **c,** An electropositive groove formed by CSN3 and CSN8 accommodates the acidic tail of CSNAP. The surfaces of CSN3 and CSN8 are coloured by electrostatic potential. **d,** CSN8 secures the C-terminus of CSNAP. **e-f,** Structural comparison between CSNAP in CSN (**e**) and DSS1, a subunit of the 26S proteasome lid complex (**f**) (PDB: 3JCK). The subunit colour code for the 26S proteasome lid corresponds to CSN.

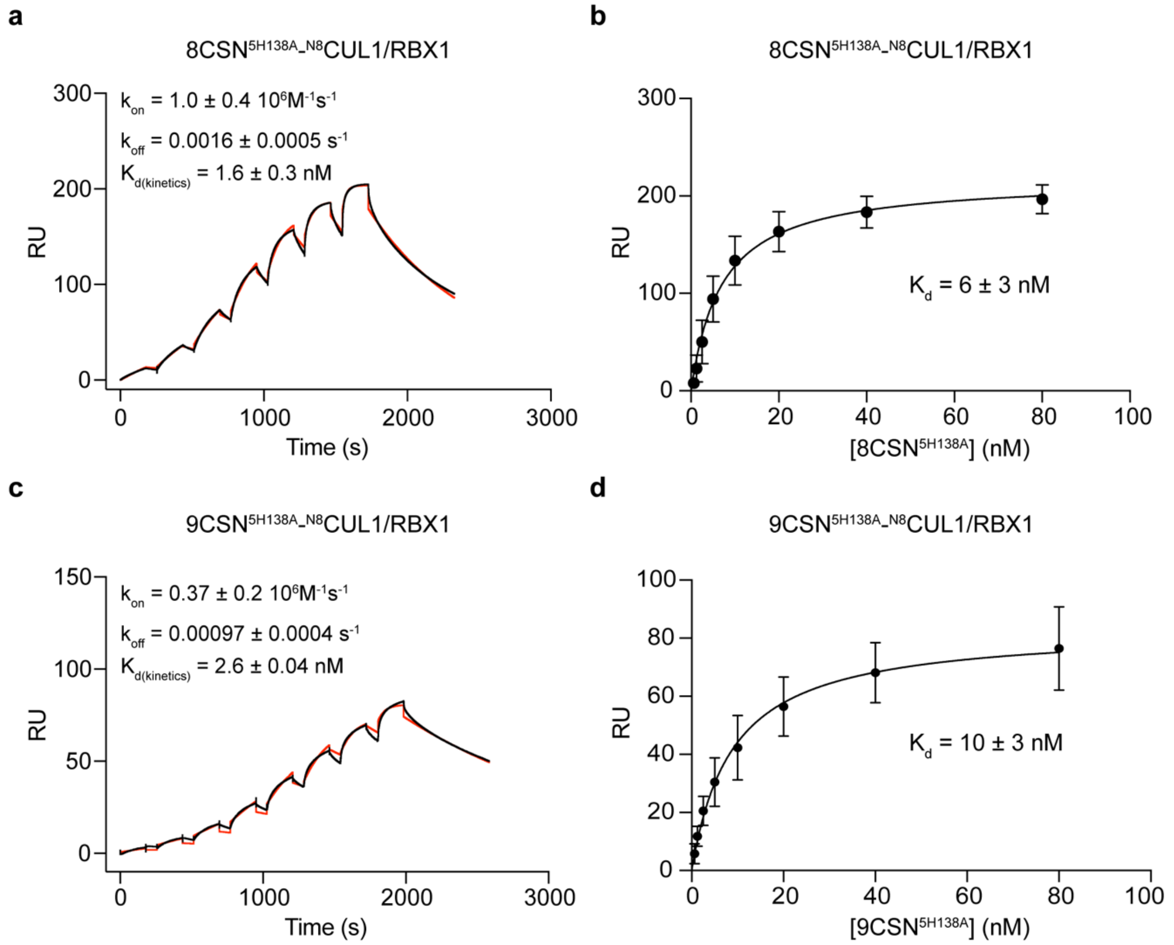
a
8CSN5H138A-N8CUL1/RBX1
kon = 1.0 ± 0.4 10^6M^-1s^-1
koff = 0.0016 ± 0.0005 s^-1
Kd(kinetics) = 1.6 ± 0.3 nM
RU
Time (s)
b
8CSN5H138A-N8CUL1/RBX1
Kd = 6 ± 3 nM
RU
[8CSN5H138A] (nM)
c
9CSN5H138A-N8CUL1/RBX1
kon = 0.37 ± 0.2 10^6M^-1s^-1
koff = 0.00097 ± 0.0004 s^-1
Kd(kinetics) = 2.6 ± 0.04 nM
RU
Time (s)
d
9CSN5H138A-N8CUL1/RBX1
Kd = 10 ± 3 nM
RU
[9CSN5H138A] (nM)

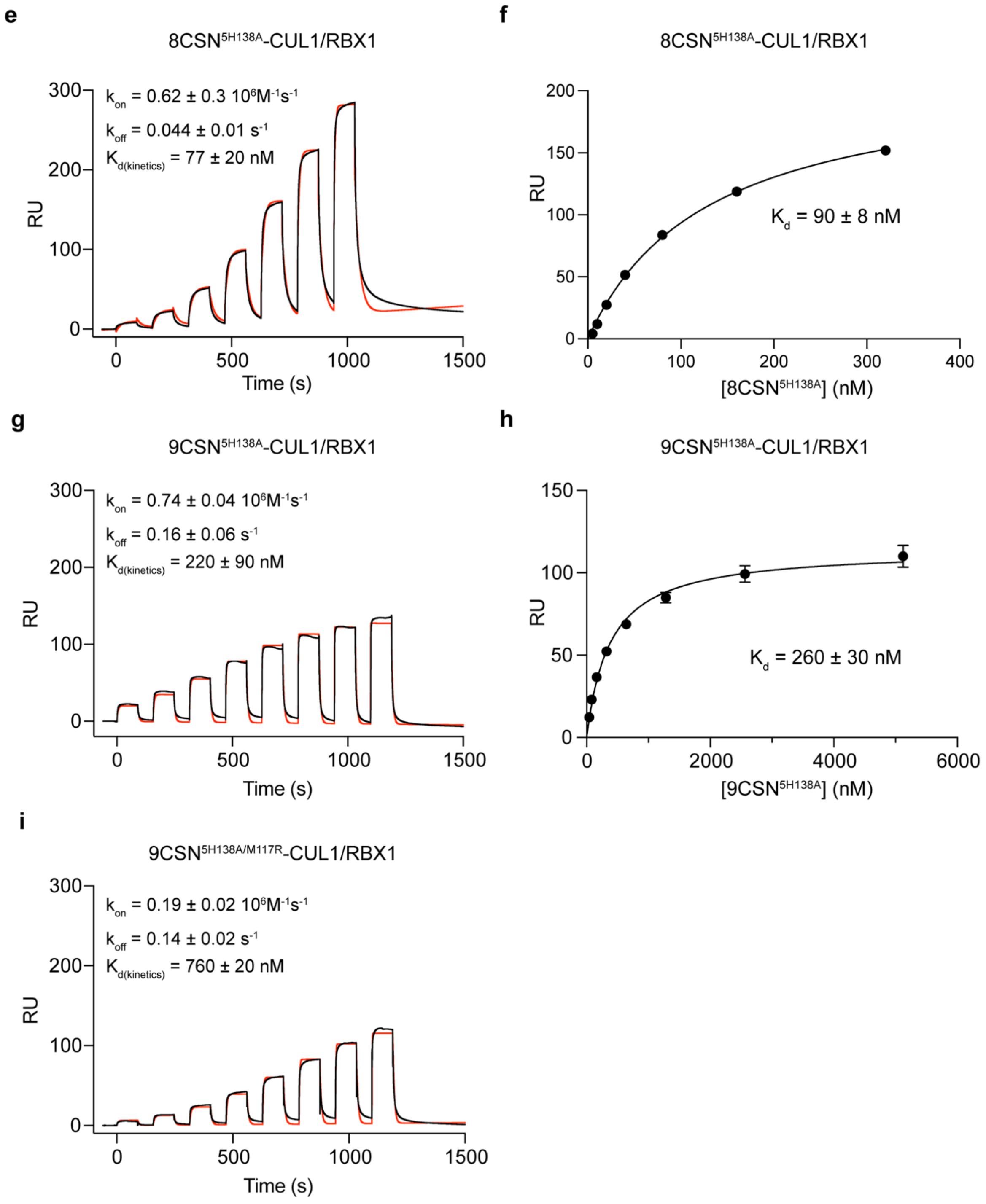

Supplementary Fig. 5. Comparative SPR analysis of CSN variants binding to neddylated and non-neddylated CUL1/RBX1

a, c, e, g, SPR sensorgrams showing binding of CSN^{5H138A} variants (with or without CSNAP) to immobilised $StrepII^{2x}$-tagged neddylated (**a, c**) or non-neddylated (**e, g**) CUL1/RBX1. Sensorgrams were analysed by global fitting using either a 1:1 Langmuir binding model (a, c) or a 1:1 binding model with drift correction (e, g), to

extract association (k_{on}) and dissociation (k_{off}) rate constants and calculate kinetic K_d values (black = experimental; red = fit). **b, d, f, h**, Corresponding steady-state binding curves derived from (**a, c, e, g**). Data were analysed by fitting a hyperbolic one-site binding model to determine steady-state K_d. For panels **b** and **f**, responses were normalised to the Rmax value to account for variability in immobilisation levels across triplicates and for the graph adjusted to the median Rmax. Raw responses were used in panels **d** and **h**. **i**, SPR sensorgrams showing binding of CSN$^{5H138A/M117R}$ to immobilised ^{StrepII2x}CUL1/RBX1. Sensorgrams were analysed by global fitting using either a 1:1 binding model with drift correction, to extract association (k_{on}) and dissociation (k_{off}) rate constants and calculate kinetic K_d values (black = experimental; red = fit). All the data represent mean ± SD from three independent experiments. Source data are available in the source data file.

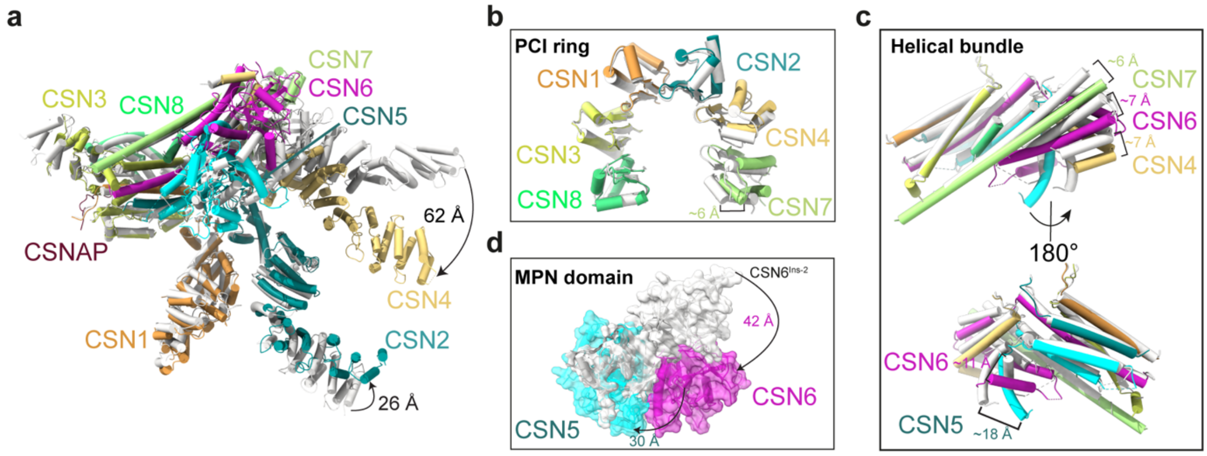

Supplementary Fig. 6. Conformational changes in CSN between the CSN^{apo} and pre-activated CSN^{5H138A} states.

a, Overall conformational changes of the CSN complex between CSN^{apo} (PDB: 4D10) (grey) and pre-activated CSN^{5H138A}-^{N8}SCF (coloured). The structural alignment was performed using CSN3 as a reference. **b,** Comparison of the CSN PCI ring architecture between CSN^{apo} (grey) and pre-activated CSN^{5H138A}-^{N8}SCF (coloured). **c,** Structural comparison of the CSN C-terminal helical bundle. **d,** Close-up view of the MPN domains of CSN5 and CSN6, illustrating conformational changes essential for CSN activation.

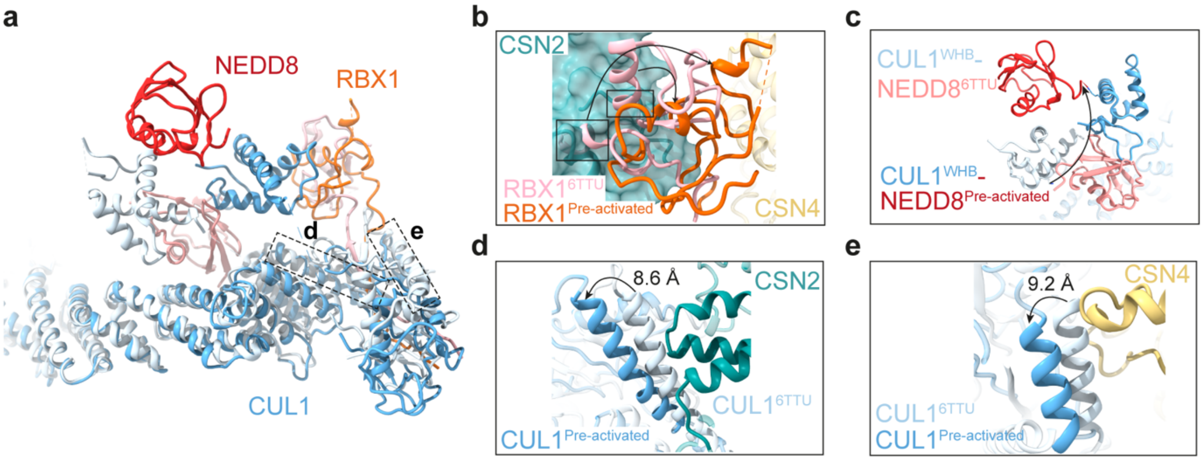

Supplementary Fig. 7. Conformational changes in ^{N8}SCF between an active ^{N8}SCF ubiquitin-bound state and the pre-activated CSN^{5H138A} complex.

a, Overall structural comparison of ^{N8}SCF between the active UBE2D2-^{N8}SCF complex (PDB: 6TTU) (grey) and pre-activated CSN^{5H138A}-^{N8}SCF (coloured) highlighting key conformational shifts. **b,** Rotation of RBX1^{RING} from its position in UBE2D2-^{N8}SCF (grey) to its new position in pre-activated CSN^{5H138A}-^{N8}SCF (coloured), preventing steric clashes with CSN2. **c,** Close-up view of conformational rearrangements in ^{N8}WHB. **d,** Structural adjustments in CUL1 to avoid a clash with CSN2. **e,** Additional conformational changes in CUL1 to avoid a clash with CSN4.

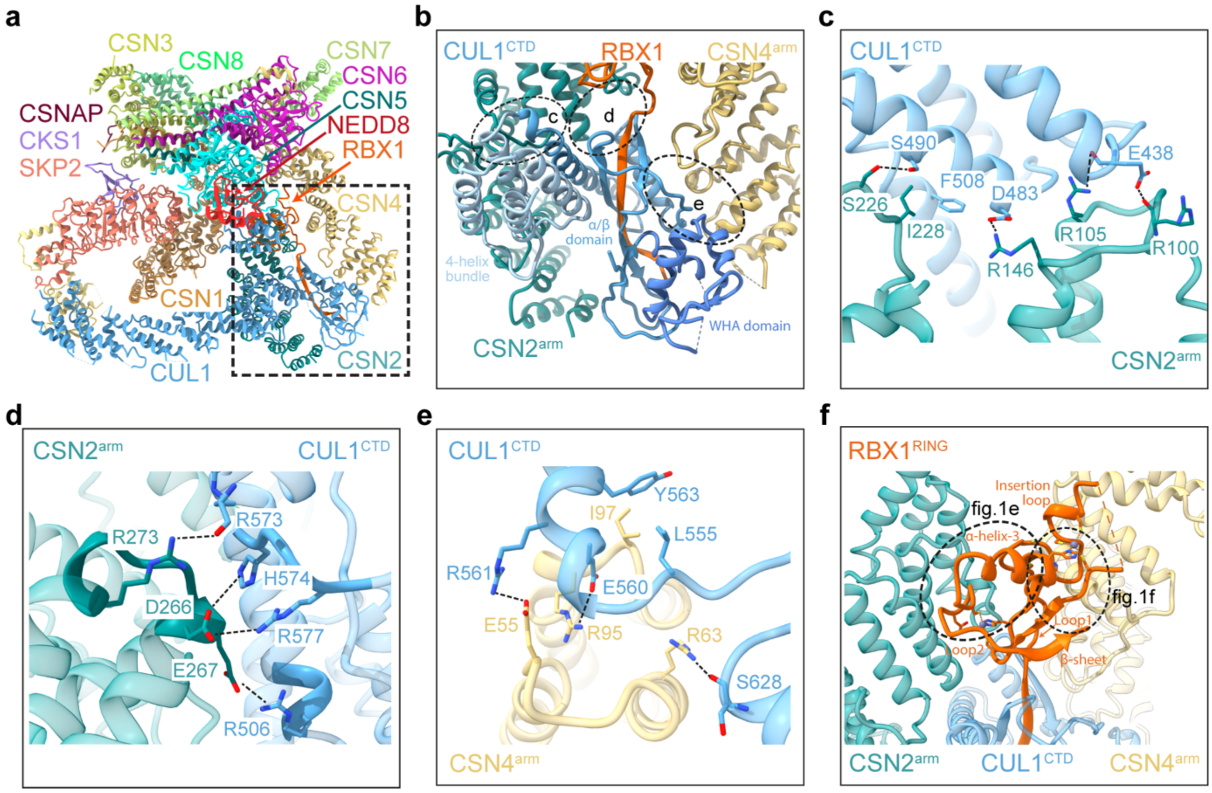

Supplementary Fig. 8. CSN2^{arm} and CSN4^{arm} clamp CUL1^{CTD} and stabilise RBX1^{RING} in pre-activated CSN^{5H138A}-^{N8}SCF.

a, Molecular model of pre-activated CSN^{5H138A}-^{N8}SCF. **b,** Close-up view of the boxed region in (**a**), illustrating how CSN2^{arm} and CSN4^{arm} clamp the CUL1^{CTD} at three distinct regions (highlighted by circles with further details in panels **c**, **d** and **e**). The 4-helix bundle (4-HB), α/β and WHA subdomains of CUL1^{CTD} are coloured in shades of blue. **c,** Detailed view of interface "c", illustrating CSN2^{arm} interactions with CUL1^{CTD}. **d,** Detailed view of interface "d", illustrating CSN2^{arm} interactions with CUL1^{CTD}. **e,** Detailed view of interface "e", showing CSN4^{arm} interactions with CUL1^{CTD}. **f,** Close-up view of the boxed region in (**a**), showing the RBX1^{RING} stabilised by CSN2^{arm} and CSN4^{arm}. Dashed circles highlight interaction interfaces related to Fig.1e and Fig.1f.

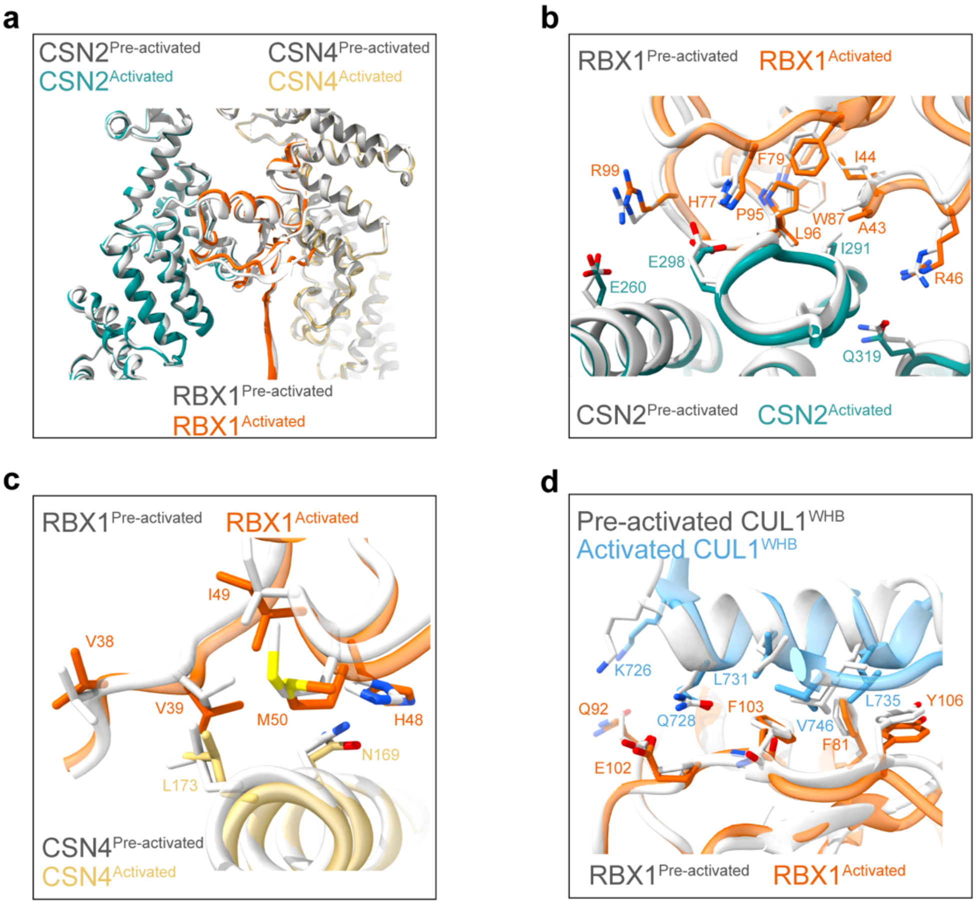

Supplementary Fig. 9. Structural comparison of pre-activated and activated CSN^{5H138A}-^{N8}SCF at the RBX1^{RING} domain

a, Superimposition of CSN2, CSN4 and the RBX1^{RING} domain from pre-activated and activated CSN^{5H138A}-^{N8}SCF structures. **b,** Close-up view comparing the CSN2-RBX1^{RING} interface in pre-activated (grey) and activated (coloured) states. **c,** Comparison of the CSN4-RBX1^{RING} interface between pre-activated (grey) and activated (coloured) states. **d,** Comparison of the CUL1^{WHB}-RBX1^{RING} interface in pre-activated (grey) and activated (coloured) states.

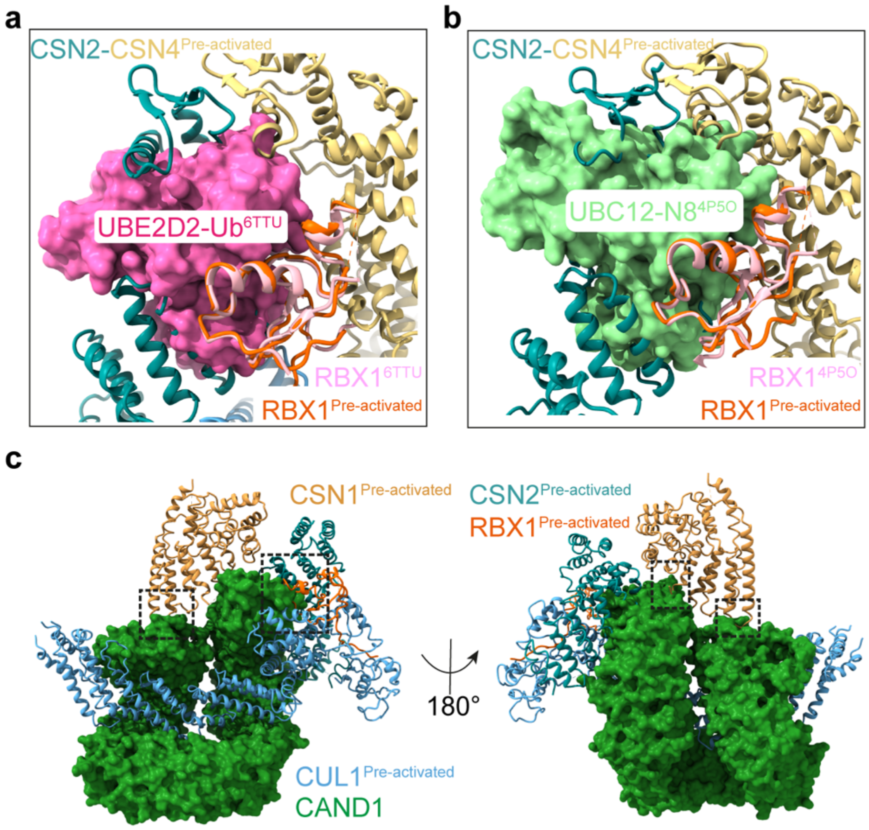

Supplementary Fig. 10. CSN directly competes with key components of the CRL regulatory cycle.

a, Structural overlay of the ubiquitin E2 (pink surface) from active UBE2D2-^{N8}SCF complex (PDB: 6TTU) with pre-activated CSN^{5H138A}-^{N8}SCF illustrating a steric clash that prevents simultaneous E2 and CSN binding. **b,** Overlay of NEDD8 E2 (green surface) from the RBX1-UBC12~^{N8}CUL1-DCNL1 complex (PDB: 4P5O) with pre-activated CSN^{5H138A}-^{N8}SCF. **c,** Overlay of CAND1 (dark green surface) from the CAND1-CUL1/RBX1-SKP1/SKP2/CKS1-CDK2 complex (PDB: 8OR0) with pre-activated CSN^{5H138A}-^{N8}SCF, revealing steric clashes between CSN1, CSN2 and RBX1 with CAND1 (highlighted by black boxes).

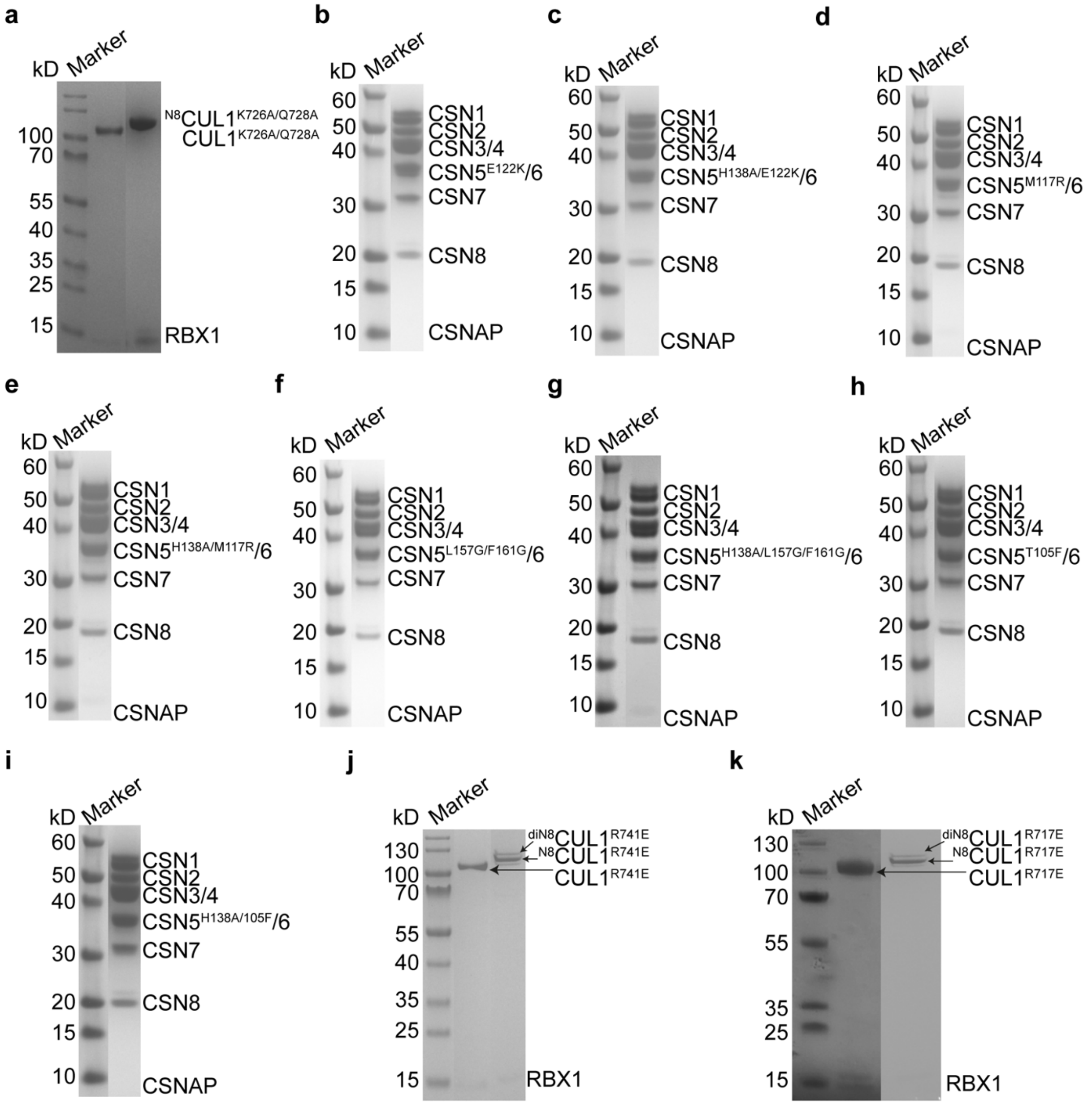

Supplementary Fig. 11. SDS-PAGE (4-12%) analysis and Coomassie staining of purified mutants.

a, Non-neddylated and neddylated CUL1$^{K726A/Q728A}$/RBX1**. b,** CSN^{5E122K}. **c,** CSN$^{5H138A/E122K}$. **d,** CSN^{5M117R}. **e,** CSN$^{5H138A/M117R}$. **f,** CSN$^{5L157G/F161G}$. **g,** CSN$^{5H138A/L157G/F161G}$. **h,** CSN^{5T105F}. **i,** CSN$^{5H138A/T105F}$. **j,** Non-neddylated and neddylated CUL1^{R741E}/RBX1**. k,** Non-neddylated and neddylated CUL1^{R717E}/RBX1.

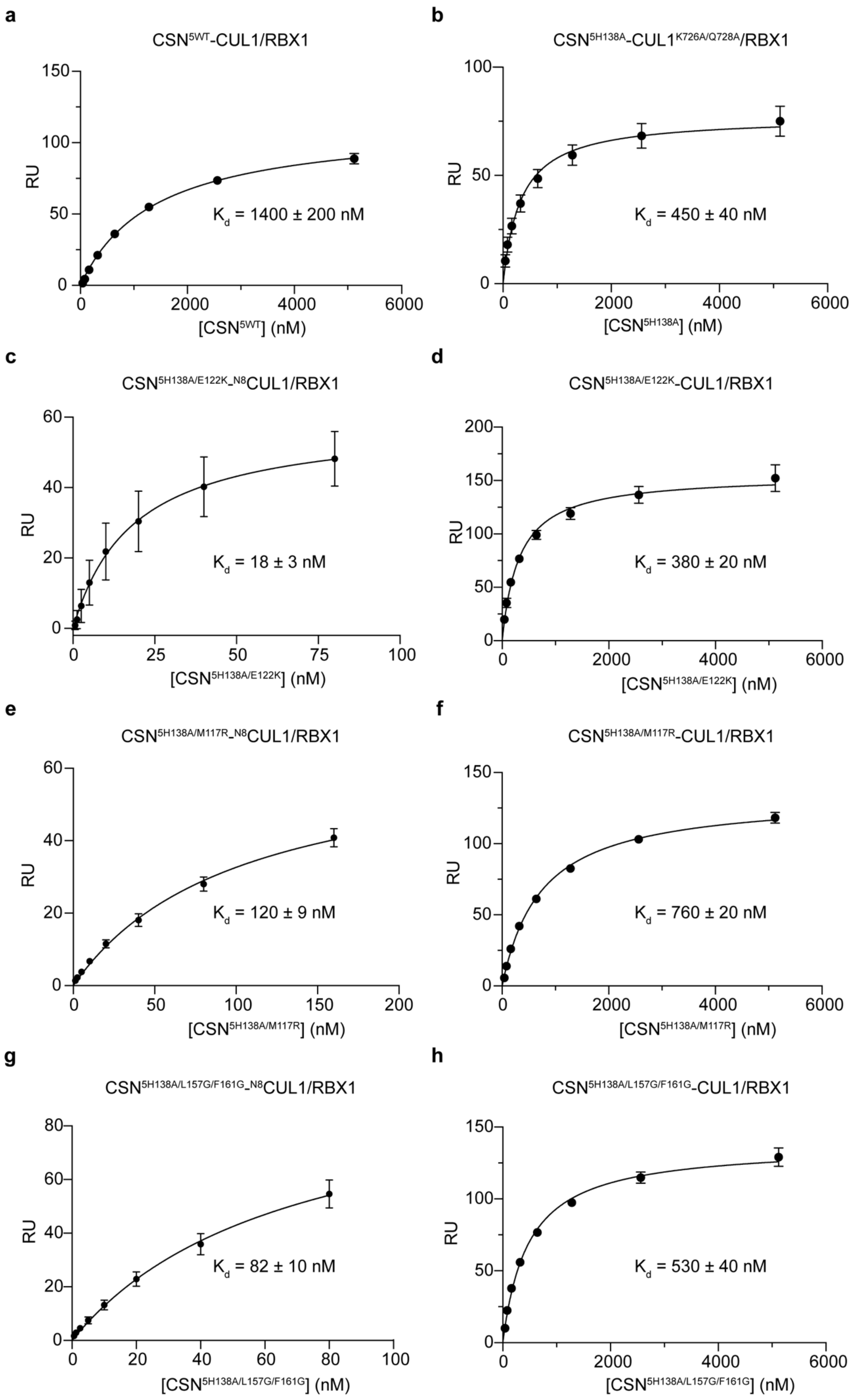
a
CSN5WT-CUL1/RBX1
RU
[CSN5WT] (nM)
Kd = 1400 ± 200 nM
b
CSN5H138A-CUL1K726A/Q728A/RBX1
RU
[CSN5H138A] (nM)
Kd = 450 ± 40 nM
c
CSN5H138A/E122K-N8CUL1/RBX1
RU
[CSN5H138A/E122K] (nM)
Kd = 18 ± 3 nM
d
CSN5H138A/E122K-CUL1/RBX1
RU
[CSN5H138A/E122K] (nM)
Kd = 380 ± 20 nM
e
CSN5H138A/M117R-N8CUL1/RBX1
RU
[CSN5H138A/M117R] (nM)
Kd = 120 ± 9 nM
f
CSN5H138A/M117R-CUL1/RBX1
RU
[CSN5H138A/M117R] (nM)
Kd = 760 ± 20 nM
g
CSN5H138A/L157G/F161G-N8CUL1/RBX1
RU
[CSN5H138A/L157G/F161G] (nM)
Kd = 82 ± 10 nM
h
CSN5H138A/L157G/F161G-CUL1/RBX1
RU
[CSN5H138A/L157G/F161G] (nM)
Kd = 530 ± 40 nM

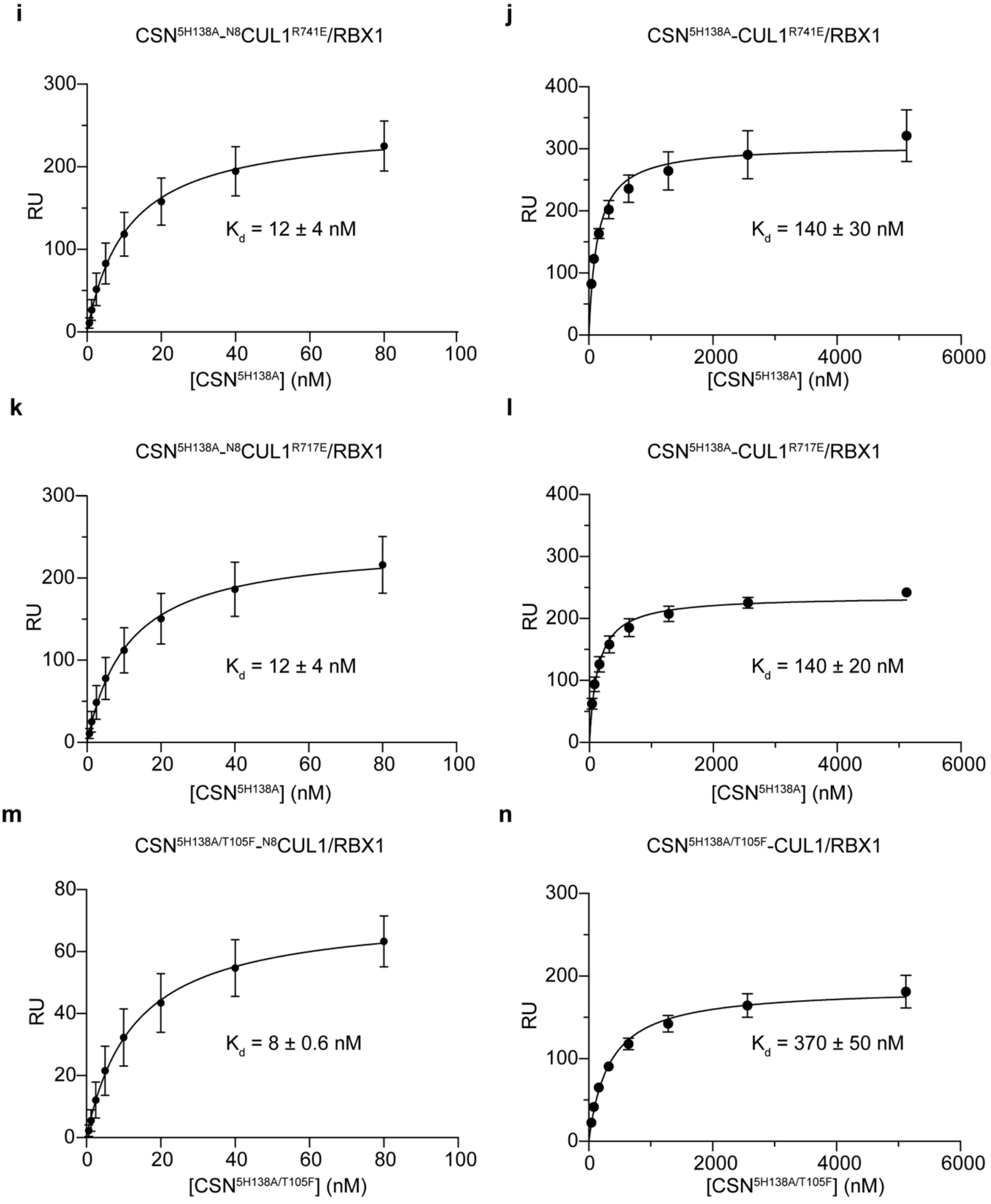
i
CSN^{5H138A}-$^{N8}CUL1^{R741E}$/RBX1
300
200
100
0
RU
K_d = 12 ± 4 nM
0 20 40 60 80 100
[CSN^{5H138A}] (nM)
j
CSN^{5H138A}-$CUL1^{R741E}$/RBX1
400
300
200
100
0
RU
K_d = 140 ± 30 nM
0 2000 4000 6000
[CSN^{5H138A}] (nM)
k
CSN^{5H138A}-$^{N8}CUL1^{R717E}$/RBX1
300
200
100
0
RU
K_d = 12 ± 4 nM
0 20 40 60 80 100
[CSN^{5H138A}] (nM)
l
CSN^{5H138A}-$CUL1^{R717E}$/RBX1
400
300
200
100
0
RU
K_d = 140 ± 20 nM
0 2000 4000 6000
[CSN^{5H138A}] (nM)
m
$CSN^{5H138A/T105F}$-^{N8}CUL1/RBX1
80
60
40
20
0
RU
K_d = 8 ± 0.6 nM
0 20 40 60 80 100
[$CSN^{5H138A/T105F}$] (nM)
n
$CSN^{5H138A/T105F}$-CUL1/RBX1
300
200
100
0
RU
K_d = 370 ± 50 nM
0 2000 4000 6000
[$CSN^{5H138A/T105F}$] (nM)

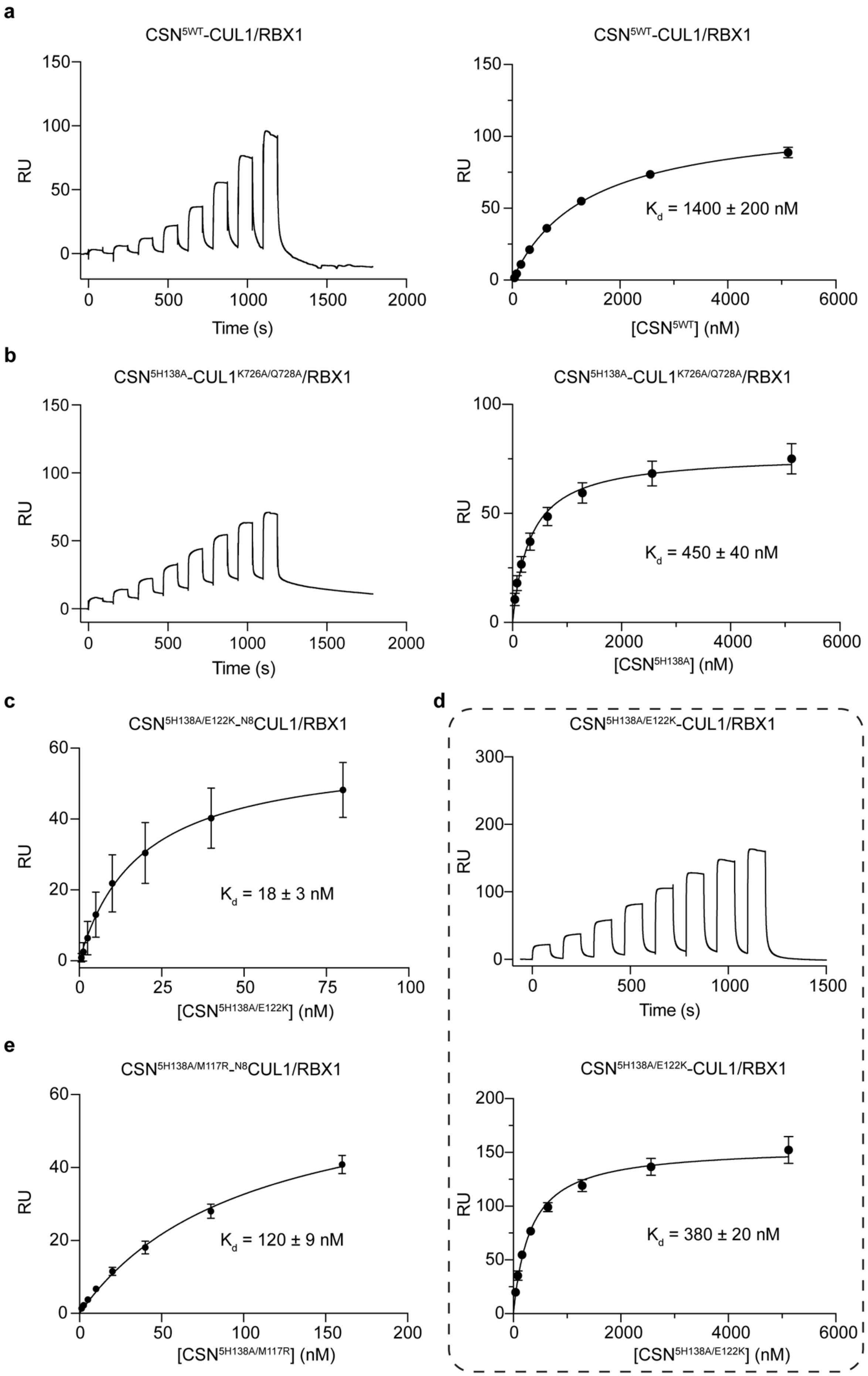
a
CSN5WT-CUL1/RBX1
RU
Time (s)
CSN5WT-CUL1/RBX1
Kd = 1400 ± 200 nM
[CSN5WT] (nM)
b
CSN5H138A-CUL1K726A/Q728A/RBX1
RU
Time (s)
CSN5H138A-CUL1K726A/Q728A/RBX1
Kd = 450 ± 40 nM
[CSN5H138A] (nM)
c
CSN5H138A/E122K-N8CUL1/RBX1
RU
Kd = 18 ± 3 nM
[CSN5H138A/E122K] (nM)
d
CSN5H138A/E122K-CUL1/RBX1
RU
Time (s)
CSN5H138A/E122K-CUL1/RBX1
Kd = 380 ± 20 nM
[CSN5H138A/E122K] (nM)
e
CSN5H138A/M117R-N8CUL1/RBX1
RU
Kd = 120 ± 9 nM
[CSN5H138A/M117R] (nM)

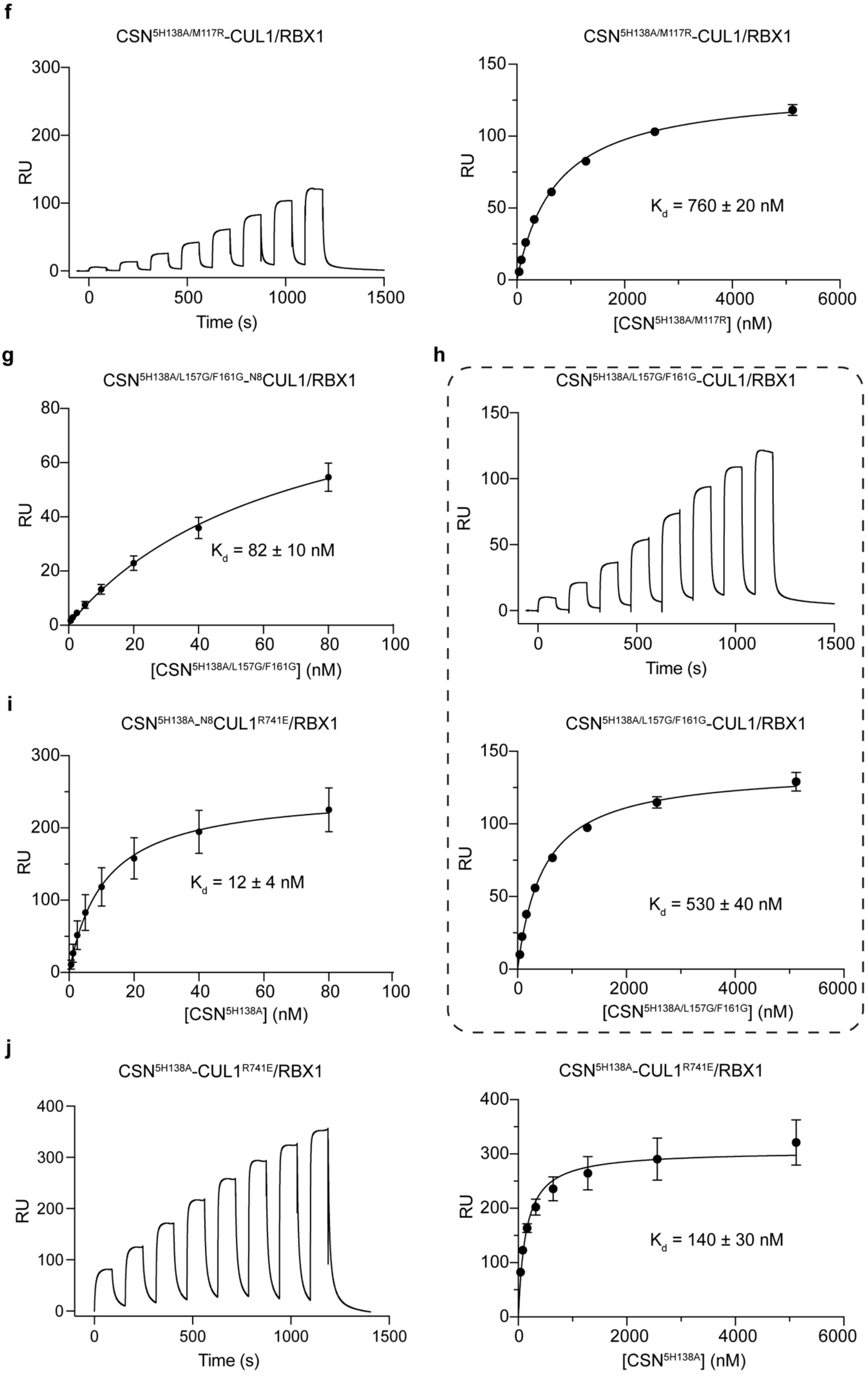

f
CSN$^{5H138A/M117R}$-CUL1/RBX1
RU
Time (s)
CSN$^{5H138A/M117R}$-CUL1/RBX1
K_d = 760 ± 20 nM
[CSN$^{5H138A/M117R}$] (nM)
g
CSN$^{5H138A/L157G/F161G}$-^{N8}CUL1/RBX1
K_d = 82 ± 10 nM
[CSN$^{5H138A/L157G/F161G}$] (nM)
h
CSN$^{5H138A/L157G/F161G}$-CUL1/RBX1
Time (s)
CSN$^{5H138A/L157G/F161G}$-CUL1/RBX1
K_d = 530 ± 40 nM
[CSN$^{5H138A/L157G/F161G}$] (nM)
i
CSN^{5H138A}-^{N8}CUL1^{R741E}/RBX1
K_d = 12 ± 4 nM
[CSN^{5H138A}] (nM)
j
CSN^{5H138A}-CUL1^{R741E}/RBX1
Time (s)
CSN^{5H138A}-CUL1^{R741E}/RBX1
K_d = 140 ± 30 nM
[CSN^{5H138A}] (nM)

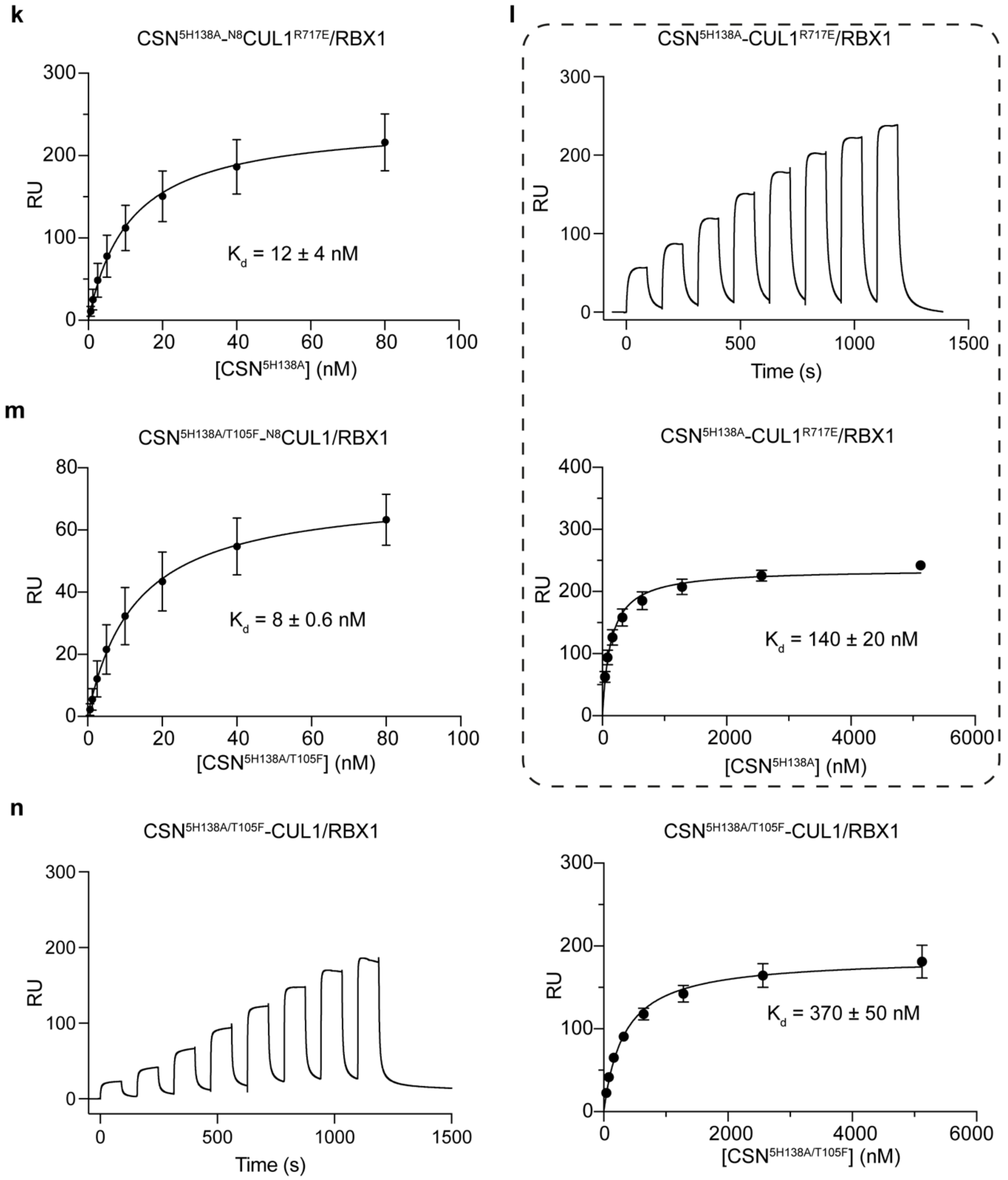

Supplementary Fig. 12. SPR analysis of binding affinities between CUL1/RBX1 and CSN mutants.

a, SPR sensorgrams (left panel) and binding curve (right panel) for affinity determination of CSN^{5WT} and ^{StrepII2x}-CUL1/RBX1. **b,** SPR sensorgrams (left panel) and binding curve (right panel) for affinity determination of CSN^{5H138A} to ^{StrepII2x}-$CUL1^{K726A/Q728A}$/RBX1. **c,** Binding curve of $CSN^{5H138A/E122K}$ and ^{StrepII2x}-^{N8}CUL1/RBX1. **d,** SPR sensorgrams (upper panel) and binding curve (bottom panel) for affinity

determination of $CSN^{5H138A/E122K}$ and ^{StrepII2x}-CUL1/RBX1. **e,** Binding curve of $CSN^{5H138A/M117R}$ and ^{StrepII2x}- ^{N8}CUL1/RBX1. **f,** SPR sensorgrams (left panel) and binding curve (right panel) for affinity determination of $CSN^{5H138A/M117R}$ and ^{StrepII2x}-CUL1/RBX1. **g,** Binding curve of $CSN^{5H138A/L157G/F161G}$ and ^{StrepII2x}- ^{N8}CUL1/RBX1. **h,** SPR sensorgrams (upper panel) and binding curve (bottom panel) for affinity determination of $CSN^{5H138A/L157G/F161G}$ and ^{StrepII2x}- CUL1/RBX1. **i,** Binding curve of CSN^{5H138A} and ^{StrepII2x}- ^{N8}CUL1^{R741E}/RBX1. **j,** SPR sensorgrams (left panel) and binding curve (right panel) for affinity determination of CSN^{5H138A} and ^{StrepII2x}-CUL1^{R741E}/RBX1. **k,** Binding curve of CSN^{5H138A} and ^{StrepII2x}- ^{N8}CUL1^{R717E}/RBX1. **l,** SPR sensorgrams (upper panel) and binding curve (bottom panel) for affinity determination of CSN^{5H138A} and ^{StrepII2x}- CUL1^{R717E}/RBX1. **m,** Binding curve of $CSN^{5H138A/T105F}$ and ^{StrepII2x}- ^{N8}CUL1/RBX1. **n,** SPR sensorgrams (left panel) and binding curve (right panel) for affinity determination of $CSN^{5H138A/T105F}$ and ^{StrepII2x}-CUL1/RBX1. Data represent the mean ± SD from three independent experiments. Dissociation constants were determined using a one-site binding hyperbola. Source data are available in the source data file.

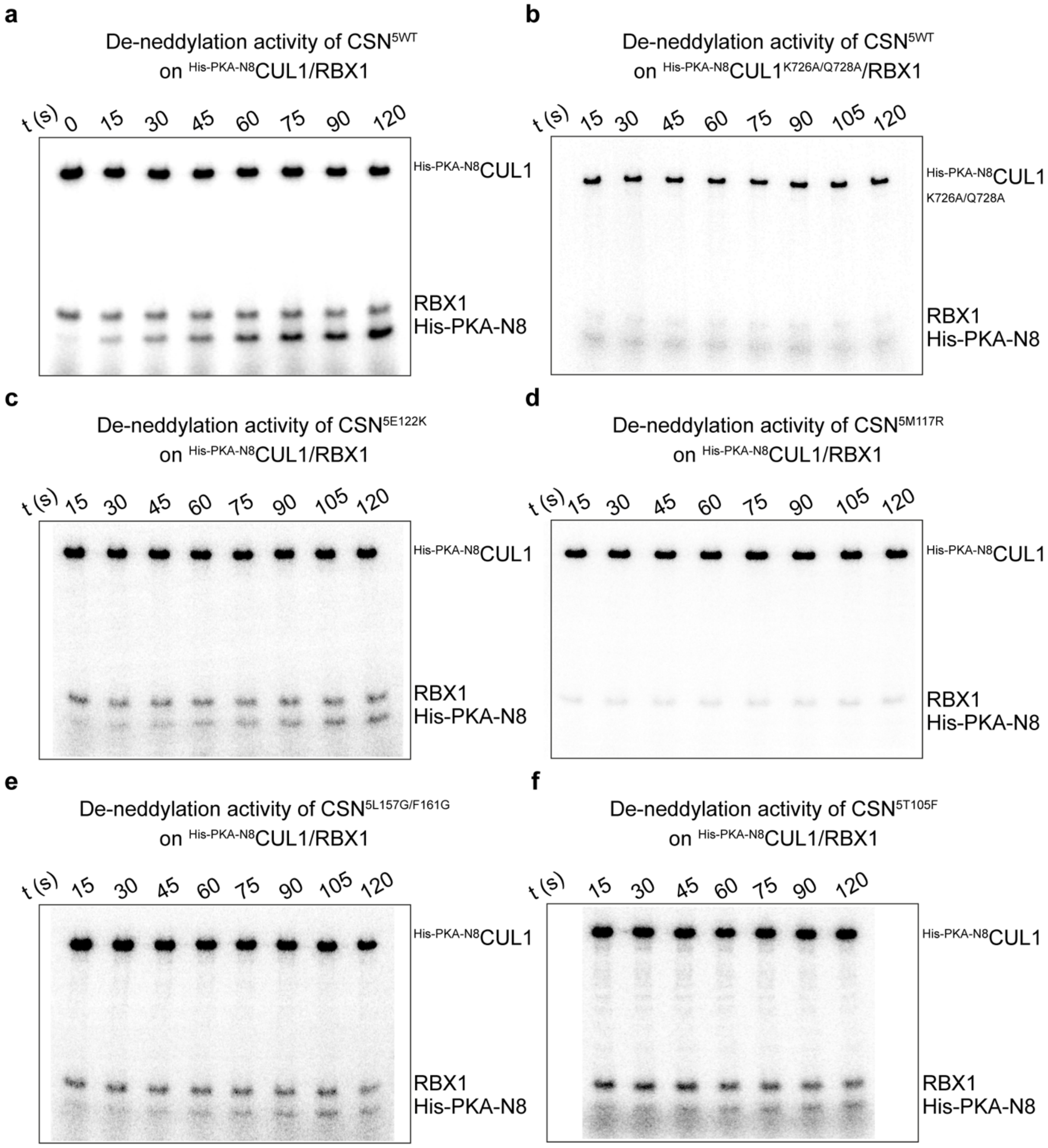
a
De-neddylation activity of CSN5WT
on His-PKA-N8CUL1/RBX1
t (s) 0 15 30 45 60 75 90 120
His-PKA-N8CUL1
RBX1
His-PKA-N8
b
De-neddylation activity of CSN5WT
on His-PKA-N8CUL1K726A/Q728A/RBX1
t (s) 15 30 45 60 75 90 105 120
His-PKA-N8CUL1
K726A/Q728A
RBX1
His-PKA-N8
c
De-neddylation activity of CSN5E122K
on His-PKA-N8CUL1/RBX1
t (s) 15 30 45 60 75 90 105 120
His-PKA-N8CUL1
RBX1
His-PKA-N8
d
De-neddylation activity of CSN5M117R
on His-PKA-N8CUL1/RBX1
t (s) 15 30 45 60 75 90 105 120
His-PKA-N8CUL1
RBX1
His-PKA-N8
e
De-neddylation activity of CSN5L157G/F161G
on His-PKA-N8CUL1/RBX1
t (s) 15 30 45 60 75 90 105 120
His-PKA-N8CUL1
RBX1
His-PKA-N8
f
De-neddylation activity of CSN5T105F
on His-PKA-N8CUL1/RBX1
t (s) 15 30 45 60 75 90 120
His-PKA-N8CUL1
RBX1
His-PKA-N8

g

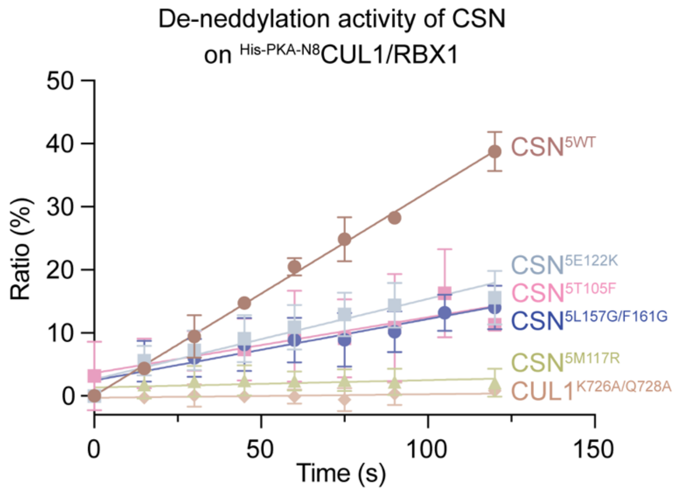

h

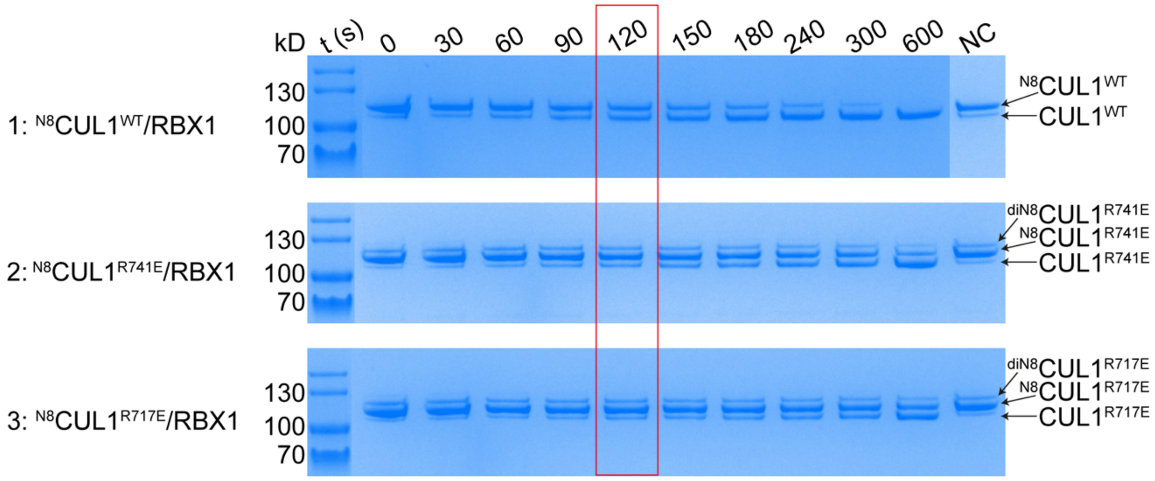

Supplementary Fig. 13. *In vitro* deneddylation activity assays.

a-f, SDS-PAGE (4–12%) analysis combined with phosphorimaging of *in vitro* deneddylation assays. The assays compare CSN5 variants, CSN^{5WT} (**a**), CSN^{5E122K} (**c**), CSN^{5M117R} (**d**), CSN$^{5L157G/F161G}$ (**e**), and CSN^{5T105F} (**f**), with $^{His\text{-}PKA\text{-}N8}$CUL1/RBX1 as substrate. Panel **b** shows CSN^{5WT} acting on a mutant substrate $^{His\text{-}PKA\text{-}N8}$CUL1K$^{726A/Q728A}$/RBX1. **g,** Quantification of deneddylation activity measured as the increasing ratio of free NEDD8 to total NEDD8 (free NEDD8 + CUL1-bound NEDD8) over time. The deneddylation activity is indicated by the slope of the linear regression curve, with mutations in CSN or CUL1 distinguished by colour-coded data points. **h,** SDS-PAGE (4-12%) and Coomassie staining of *in vitro* deneddylation assays comparing 1: ^{N8}CUL1/RBX1; 2: ^{N8}CUL1^{R741E}/RBX1; and 3: ^{N8}CUL1^{R717E}/RBX1.

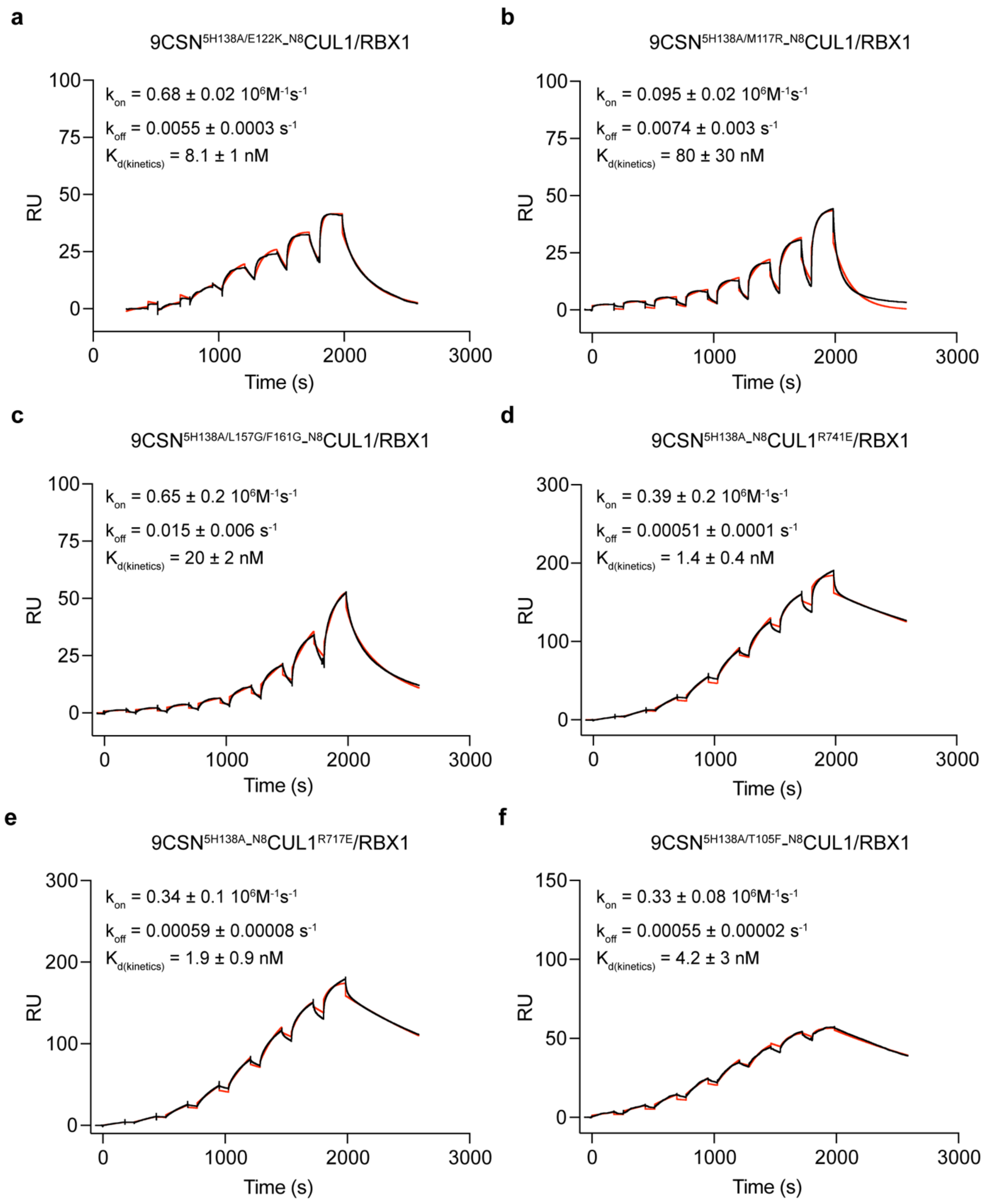

Supplementary Fig. 14. SPR kinetic analysis of CSN variants binding to neddylated CUL1/RBX1 variants

SPR sensorgrams showing binding of CSN$^{5H138A/E122K}$ (**a**), CSN$^{5H138A/M117R}$ (**b**), CSN$^{5H138A/L157G/F161G}$ (**c**), CSN$^{5H138A/T105F}$ (**f**) to immobilised StrepII^{2x}-tagged neddylated CUL1/RBX1. Sensorgrams showing binding of CSN^{5H138A} to mutated

$^{StrepII2x\text{-}N8}$CUL1^{R741E}/RBX1(**d**), and $^{StrepII2x\text{-}N8}$CUL1^{R717E}/RBX1 (**e**). Sensorgrams were globally fitted using a 1:1 Langmuir binding model (black = experimental; red = fit) to extract association (k_{on}) and dissociation (k_{off}) rate constants and calculate kinetic dissociation constants (K_d). Data represent mean ± SD from three independent experiments. Source data are available in the source data file.

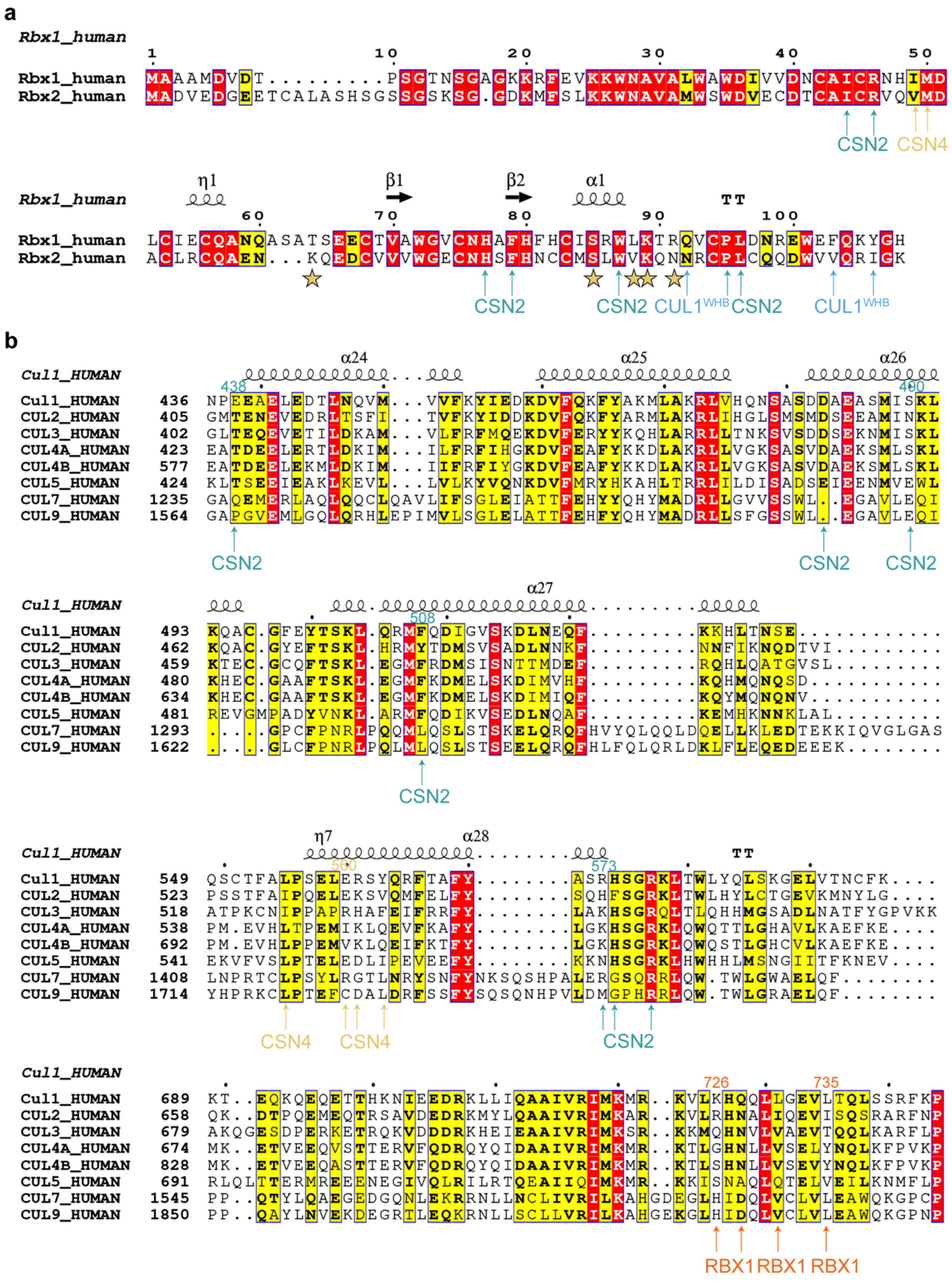
a
b
Rbx1_human
Rbx2_human
CSN2
CSN4
CUL1WHB
Cul1_HUMAN
CUL2_HUMAN
CUL3_HUMAN
CUL4A_HUMAN
CUL4B_HUMAN
CUL5_HUMAN
CUL7_HUMAN
CUL9_HUMAN
RBX1

Supplementary Fig. 15. Conservation of key interaction residues in CUL1 and RBX1.

a, Sequence alignment of human RBX1 and RBX2, highlighting key residues involved in interactions with CSN2 (green arrows), CSN4 (yellow arrows) and CUL^{WHB} (blue arrows). Residues specifically contributing to CSN4 interactions in dissociation-state-3 are marked with a yellow star. **b,** Sequence alignment of the human CUL1 family, with key residues mediating interactions with CSN2 (green arrows), CSN4 (yellow arrows) and RBX1 (orange arrows).

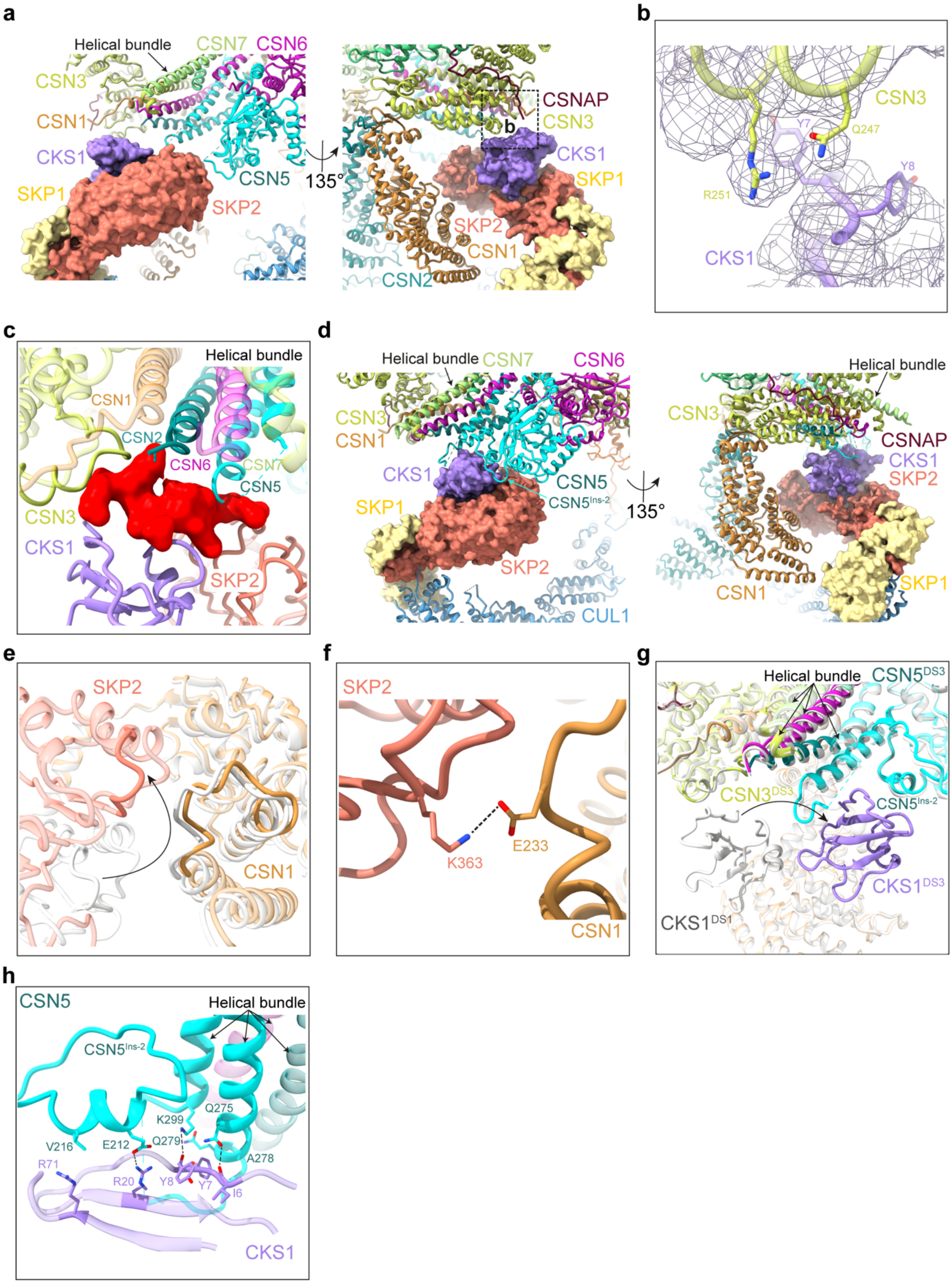

Supplementary Fig. 16. Structural analysis of SR recognition by CSN.

a, Location of the SR in pre-activated CSN^{5H138A} -^{N8}SCF. SKP2 and CKS1 are positioned within a space formed by CSN1, CSN3, the CSN helical bundle, and

CSN5. CSN subunits are displayed in ribbon, while the SR subunits are represented as a surface. **b,** In pre-activated CSN^{5H138A} -^{N8}SCF, the SR subunit CKS1 appears to be stabilised by a small interface with CSN3, as supported by the cryo-EM density shown as a mesh. **c,** Docking of p27 (PDB: 2AST), the substrate of SKP1-CKS1, onto pre-activated CSN^{5H138A} -^{N8}SCF, with p27 shown as a red surface. The model suggests that CSN cannot engage with the active SCF, as the presence of the substrate induces steric clashes with CSN. **d,** Location of the SR in CSN^{E104A}-SCF dissociation-state-3. Here, the SR occupies a space defined by the $CSN1^{arm}$, the CSN5 helical bundle, and the CSN5 MPN domain. CSN subunits are depicted in ribbon, while SR subunits are represented as a surface. **e,** Structural overlay highlighting key conformational changes in SKP2 between CSN^{E104A}-SCF dissociation-state-1 (grey) and CSN^{E104A}-SCF dissociation-state-3 (coloured), illustrating its structural rearrangement. **f,** Small but notable interface between SKP2 and CSN1 in CSN^{E104A}-SCF dissociation-state-3. **g,** Structural overlay highlighting key conformational changes in CKS1 between CSN^{E104A}-SCF dissociation-state-1 (grey) and CSN^{E104A}-SCF dissociation-state-3 (coloured). **h,** In CSN^{E104A}-SCF dissociation-state-3, there is an extensive interaction interface between CKS1, $CSN5^{Ins\text{-}2}$ and the CSN5 helical bundle.

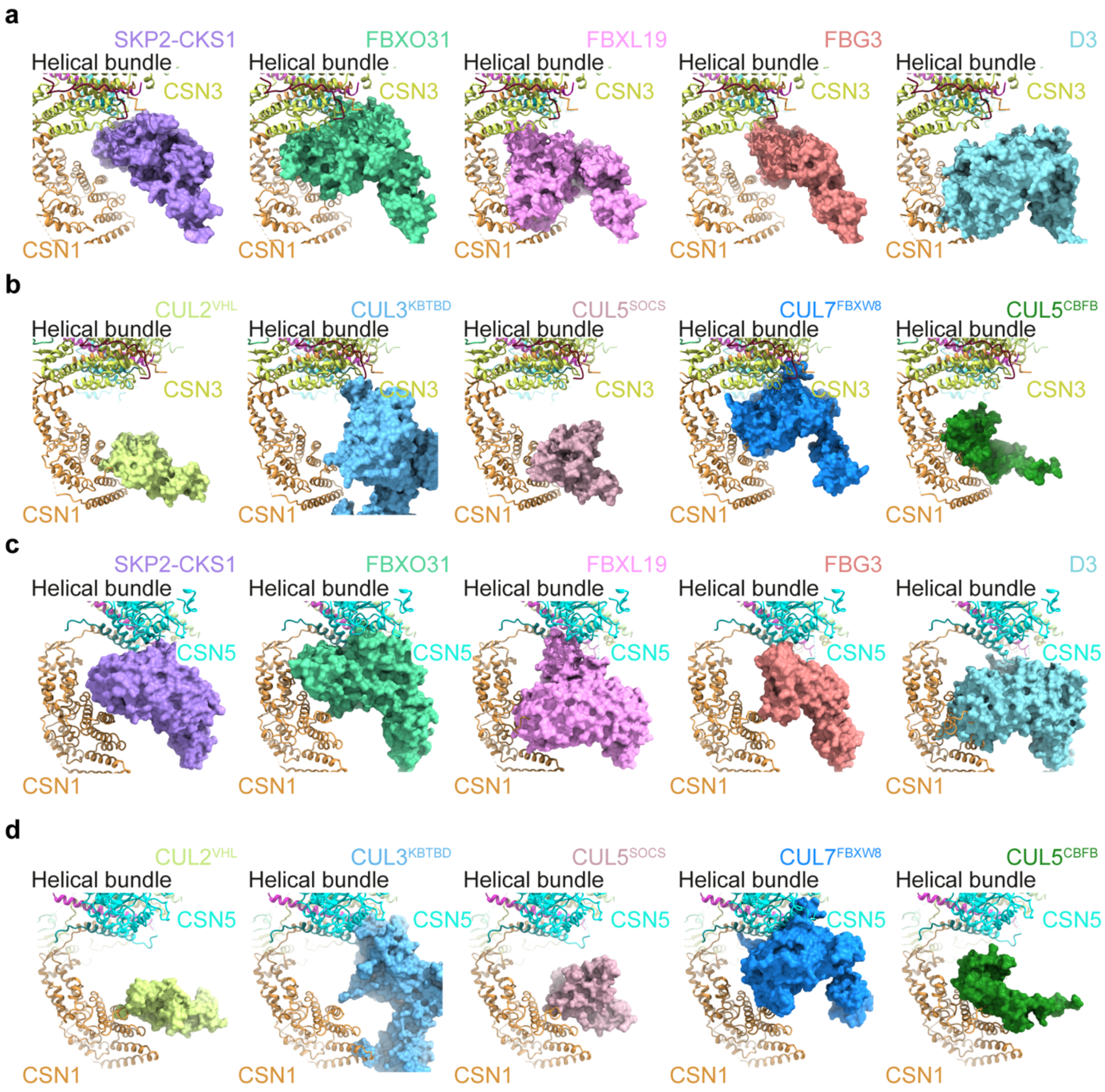

Supplementary Fig. 17. Structural docking of diverse SRs onto pre-activated CSN^{5H138A} -^{N8}SCF and CSN^{E104A}-SCF dissociation-state-3.

a, Docking of various F-box SRs onto pre-activated CSN^{5H138A} -^{N8}SCF reveals a conserved positioning, with the docked SRs aligning similarly to SKP2 in pre-activated CSN^{5H138A} -^{N8}SCF. The PDB codes for the docked SRs are: FBXO31 (5VZT), FBXL19 (6WCQ), FBG3 (3WSO), and D3 (6BRO). **b**, Docking of SRs from different CRLs onto pre-activated CSN^{5H138A} -^{N8}SCF demonstrates CSNs adaptability through an induced-fit mechanism, accommodating a broad range of SRs with distinct sizes and geometries. The PDB codes for the docked SRs are: CUL2^{VHL} (4WQO), CUL3^{KBTBD} (8H38), CUL5^{SOCS} (4JGH), CUL7^{FBXW8} (7Z8B), and CUL5^{CBFB}

(4N9F). **c,** Similar docking analysis of F-box protein SRs onto CSN^{E104A}-SCF dissociation-state-3 as described in (**a**). **d**, Docking of SRs from different CRLs onto CSN^{E104A}-SCF dissociation-state-3 as described in (**a**), highlights an additional role for CSN5 in SR recognition during later dissociation states.

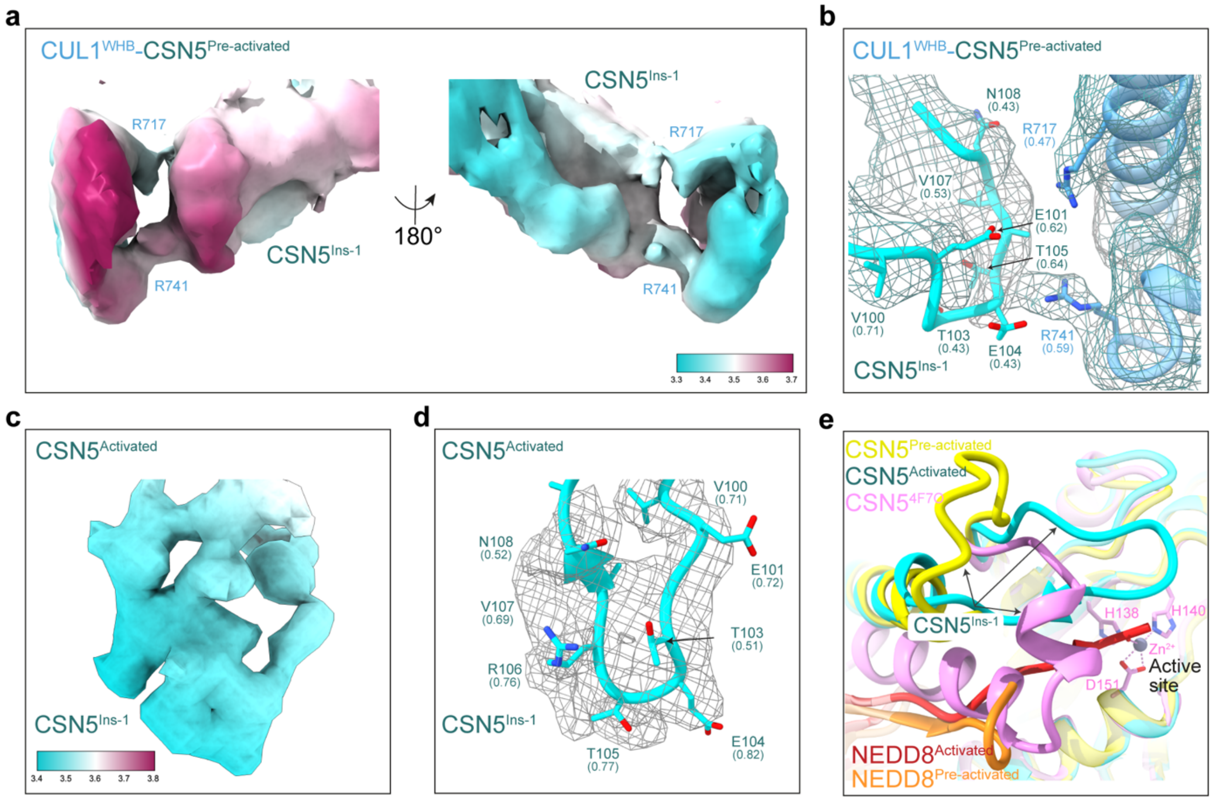

Supplementary Fig. 18. Structural versatility of CSN5$^{Ins\text{-}1}$.

a, Local resolution estimation of CSN5$^{Ins\text{-}1}$ and CUL1^{R741}, CUL1^{R717} in pre-activated CSN^{5H138A}-^{N8}SCF. **b,** Cryo-EM density and molecular model of CSN5$^{Ins\text{-}1}$, as well as CUL1^{R741} and CUL1^{R717} in pre-activated CSN^{5H138A}-^{N8}SCF. The side chains of CSN5^{E101} and CSN5^{E104} are modelled as approximations due to density limitations. For validation, the Q-score [5] is calculated for the backbone and quoted under each residue. **c,** Local resolution estimation of CSN5$^{Ins\text{-}1}$ in activated CSN^{5H138A}-^{N8}SCF. **d,** Cryo-EM density and molecular model of CSN5$^{Ins\text{-}1}$ in activated CSN^{5H138A}-^{N8}SCF. **e,** Structural comparison of CSN5$^{Ins\text{-}1}$ from pre-activated and activated CSN^{5H138A}-^{N8}SCF, alongside the isolated crystal structure of CSN5 (PDB: 4F7O), demonstrating the conformational adaptability of this loop.

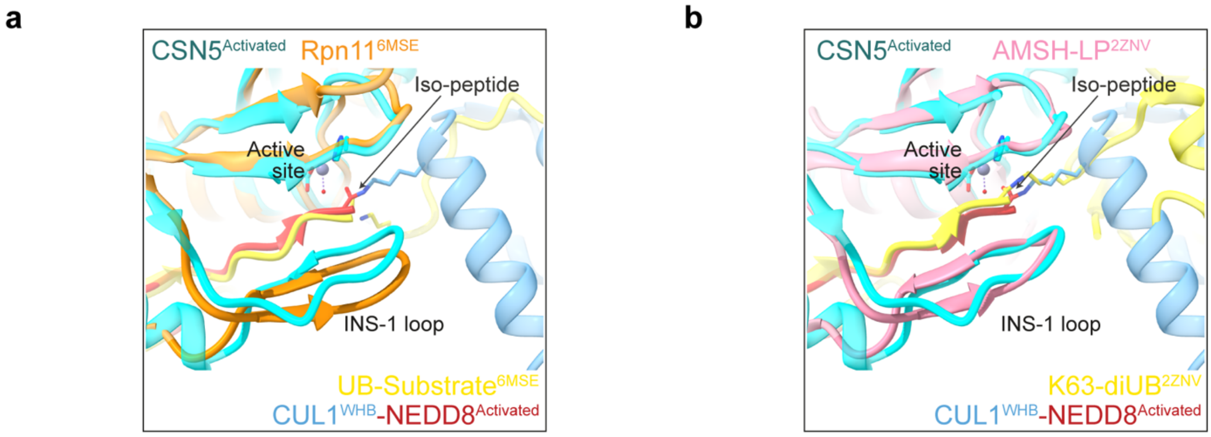

Supplementary Fig. 19. Structural comparison of active sites in activated CSN^{5H138A}-^{N8}SCF and MPN^{+} metalloprotease family members.

a, Structural alignment of CSN5, NEDD8 and CUL1^{WHB} in activated CSN^{5H138A}-^{N8}SCF with the RPN11-Ub from the 26S proteasome complex (PDB: 6MSE). **b,** Structural alignment of CSN5, and NEDD8 and CUL1^{WHB} in activated CSN^{5H138A}-^{N8}SCF with the AMSH-LP-diUb complex (PDB: 2ZNV). In both cases, the conserved Ins-1 loop adopts a β-hairpin motif, stabilising the β-stranded C-terminal ubiquitin tail. However, in activated CSN^{5H138A}-^{N8}SCF, while CSN5^{Ins-1} similarly stabilises the CUL1^{K720}-NEDD8^{G76} isopeptide bond, it does not adopt a β-hairpin structure.

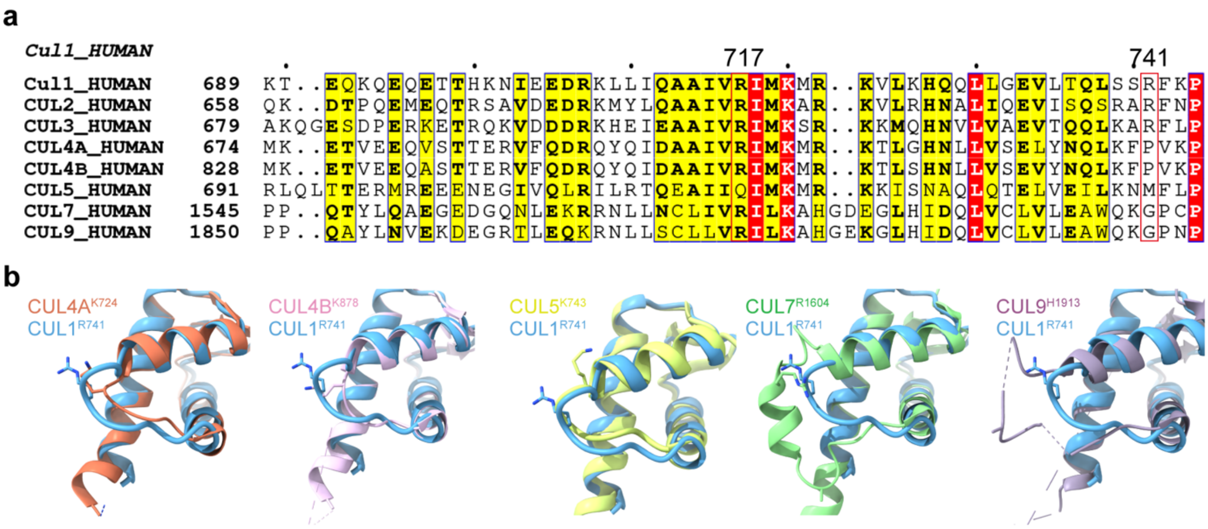

Supplementary Fig. 20. Conservation analysis of R717 and R741 across the Cullin family.

a, Sequence alignment of the human Cullin family, highlighting $CUL1^{R717}$ and $CUL1^{R741}$ (red boxes). Sequence alignment was performed using the MUSCLE algorithm in Snapgene [6]. **b,** Structural alignment of $CUL1^{R741}$ with CUL4A, CUL4B, CUL5, CUL7 and CUL9, demonstrating the structural conservation of $CUL1^{R741}$. PDB codes for respective cullins are as follows: CUL4A: 2HYE [7]; CULB: 4A0L [8]; CUL5: 3DQV [9]; CUL7: 7Z8B [10]; CUL9: 8Q7H [11].

a **b**

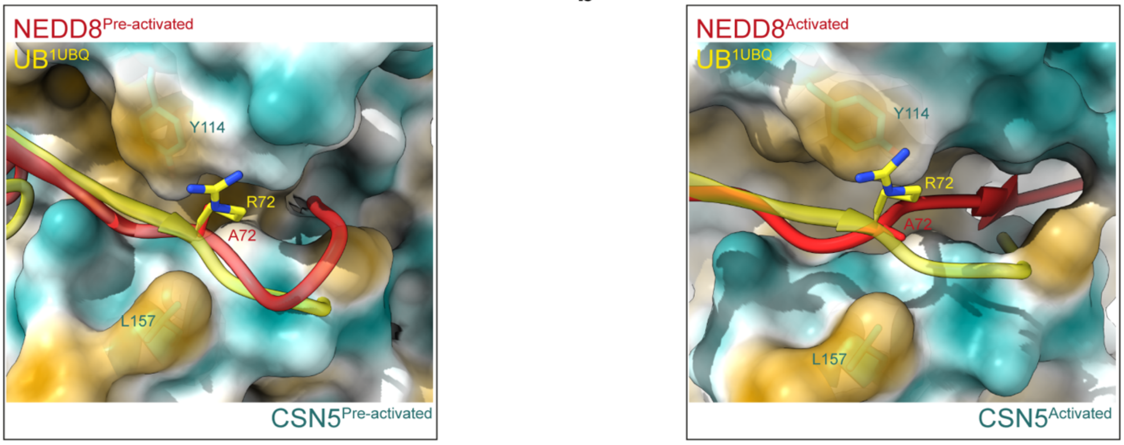

Supplementary Fig. 21. CSN5 exhibits strict selectivity for NEDD8.

a, Structural overlay of Ubiquitin (Ub) (PDB: 1UBQ) onto NEDD8 in pre-activated CSN^{5H138A}-^{N8}SCF. CSN5 is displayed as a hydrophobic surface. **b,** Structural overlay of Ubiquitin (PDB: 1UBQ) onto NEDD8 in activated CSN^{5H138A}-^{N8}SCF. The bulky charged Ub^{R72} side chain is poorly accommodated within CSN5's hydrophobic pocket.

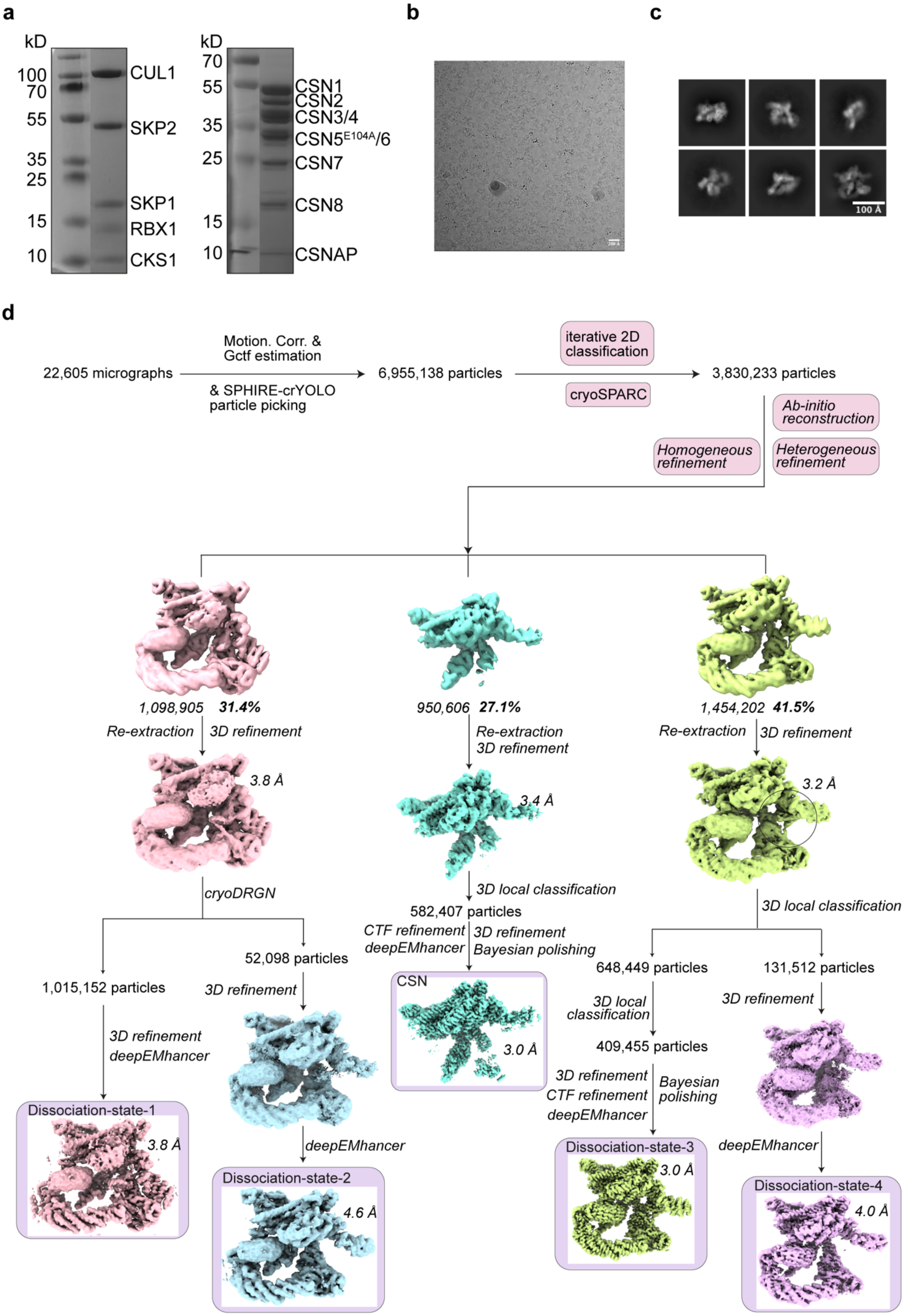
a
kD
100
70
55
35
25
15
10
CUL1
SKP2
SKP1
RBX1
CKS1
kD
70
55
35
25
15
10
CSN1
CSN2
CSN3/4
CSN5^E104A/6
CSN7
CSN8
CSNAP
b
c
100 Å
d
22,605 micrographs
Motion. Corr. &
Gctf estimation
& SPHIRE-crYOLO
particle picking
6,955,138 particles
iterative 2D
classification
cryoSPARC
3,830,233 particles
Ab-initio
reconstruction
Homogeneous
refinement
Heterogeneous
refinement
1,098,905 31.4%
Re-extraction
3D refinement
3.8 Å
cryoDRGN
1,015,152 particles
3D refinement
deepEMhancer
Dissociation-state-1
3.8 Å
52,098 particles
3D refinement
deepEMhancer
Dissociation-state-2
4.6 Å
950,606 27.1%
Re-extraction
3D refinement
3.4 Å
3D local classification
582,407 particles
CTF refinement
deepEMhancer
3D refinement
Bayesian polishing
CSN
3.0 Å
1,454,202 41.5%
Re-extraction
3D refinement
3.2 Å
3D local classification
648,449 particles
3D local
classification
409,455 particles
3D refinement
CTF refinement
deepEMhancer
Bayesian
polishing
Dissociation-state-3
3.0 Å
131,512 particles
3D refinement
deepEMhancer
Dissociation-state-4
4.0 Å

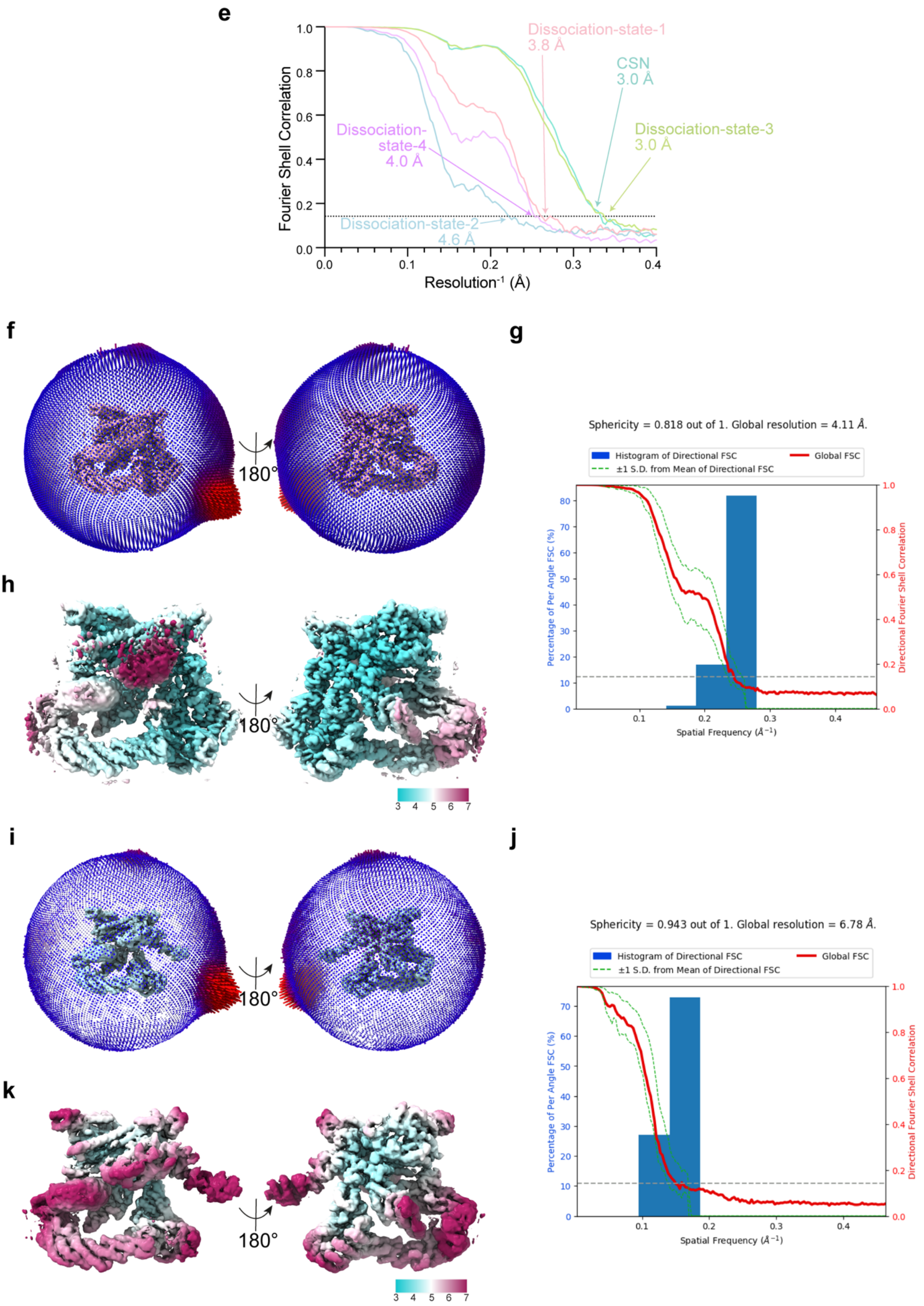
e
Fourier Shell Correlation
Resolution⁻¹ (Å)
Dissociation-state-1
3.8 Å
CSN
3.0 Å
Dissociation-state-3
3.0 Å
Dissociation-
state-4
4.0 Å
Dissociation-state-2
4.6 Å
f
180°
g
Sphericity = 0.818 out of 1. Global resolution = 4.11 Å.
Histogram of Directional FSC
±1 S.D. from Mean of Directional FSC
Global FSC
Percentage of Per Angle FSC (%)
Directional Fourier Shell Correlation
Spatial Frequency (Å⁻¹)
h
180°
i
180°
j
Sphericity = 0.943 out of 1. Global resolution = 6.78 Å.
Histogram of Directional FSC
±1 S.D. from Mean of Directional FSC
Global FSC
Percentage of Per Angle FSC (%)
Directional Fourier Shell Correlation
Spatial Frequency (Å⁻¹)
k
180°

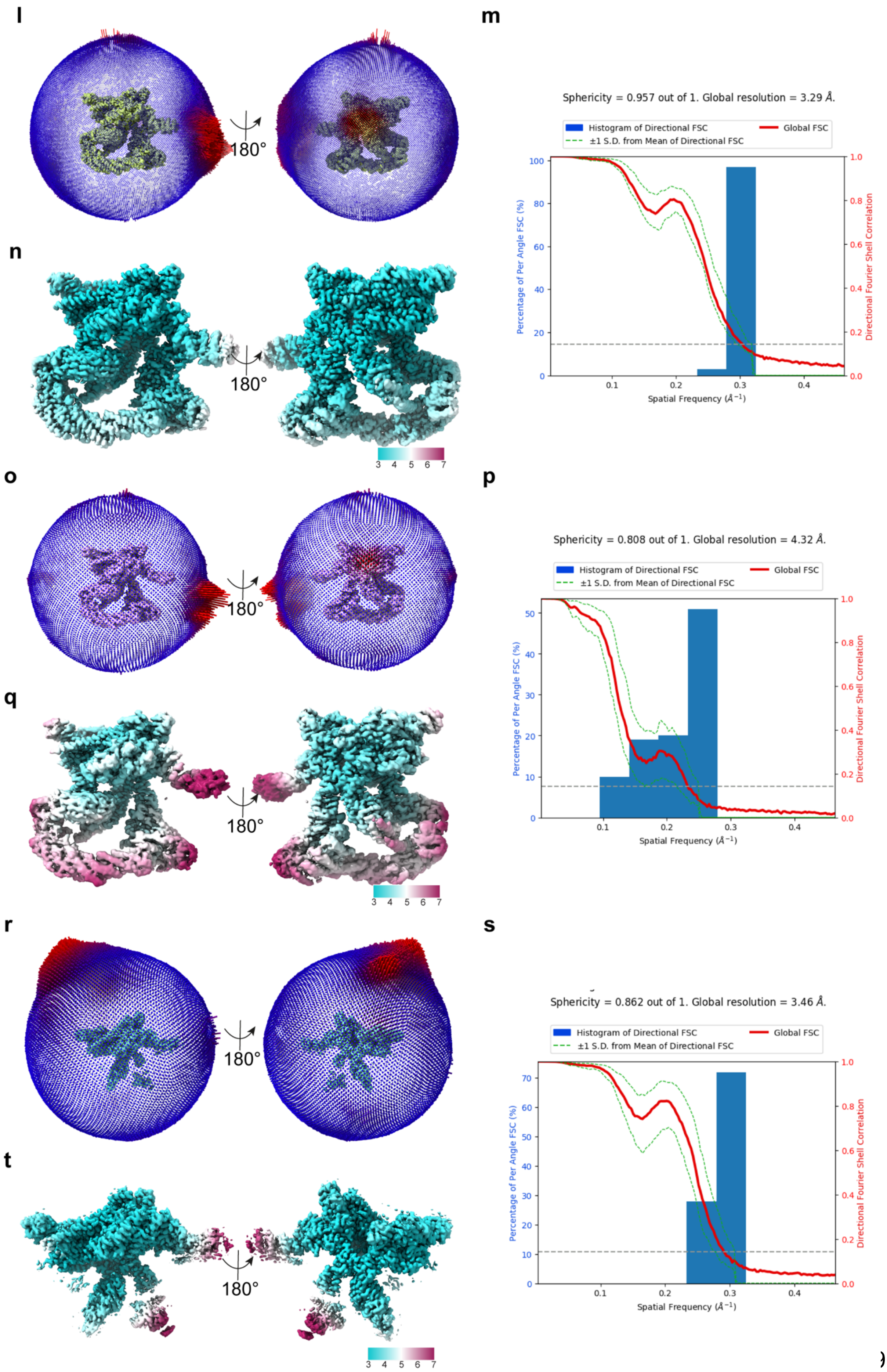

l
m
180°
Sphericity = 0.957 out of 1. Global resolution = 3.29 Å.
Histogram of Directional FSC
Global FSC
±1 S.D. from Mean of Directional FSC
Percentage of Per Angle FSC (%)
Directional Fourier Shell Correlation
Spatial Frequency (Å^{-1})
n
180°
3 4 5 6 7
o
p
180°
Sphericity = 0.808 out of 1. Global resolution = 4.32 Å.
Histogram of Directional FSC
Global FSC
±1 S.D. from Mean of Directional FSC
Percentage of Per Angle FSC (%)
Directional Fourier Shell Correlation
Spatial Frequency (Å^{-1})
q
180°
3 4 5 6 7
r
s
180°
Sphericity = 0.862 out of 1. Global resolution = 3.46 Å.
Histogram of Directional FSC
Global FSC
±1 S.D. from Mean of Directional FSC
Percentage of Per Angle FSC (%)
Directional Fourier Shell Correlation
Spatial Frequency (Å^{-1})
t
180°
3 4 5 6 7

Supplementary Fig. 22. Cryo-EM and single particle analysis on CSN^{5E104A}-SCF dissociation states.

a, SDS-PAGE (4-12%) analysis and Coomassie staining of assembled SCF complex (CUL1, RBX1, SKP1, SKP2 and CKS1) and reconstituted CSN5^{E104A}. **b,** A representative motion-corrected micrograph and particle picking (red circles). **c,** Representative high-quality 2D reference-free 2D class averages. **d,** Single particle analysis workflow for the CSN^{5E104A}-SCF dataset. 3D classifications were performed in CryoDRGN [2] and RELION-4.0 [1]). **e,** Resolution estimates of maps resolved from the CSN^{E104A}-SCF dataset. **f,** Euler angle distribution plots for dissociation-state-1. **g,** Directional 3DFSC plots of dissociation-state-1. **h,** Local resolution estimates for dissociation-state-1. **i,** Euler angle distribution plots for dissociation-state-2. **j,** Directional 3DFSC plots of dissociation-state-2. **k,** Local resolution estimates of dissociation-state-2. **l,** Euler angle distribution plots for dissociation-state-3. **m,** Directional 3DFSC plots of dissociation-state-3. **n,** Local resolution estimates of dissociation-state-3. **o,** Euler angle distribution plots of dissociation-state-4. **p,** Directional 3DFSC plots of dissociation-state-4. **q,** Local resolution estimates of dissociation-state-4. **r,** Euler angle distribution plots of free CSN (CSN^{Apo}). **s,** Directional 3DFSC plots of free CSN (CSN^{Apo}). **t,** Local resolution estimates of free CSN (CSN^{Apo}). Resolutions for all maps in this figure were estimated using the gold-standard FSC 0.143 criterion.

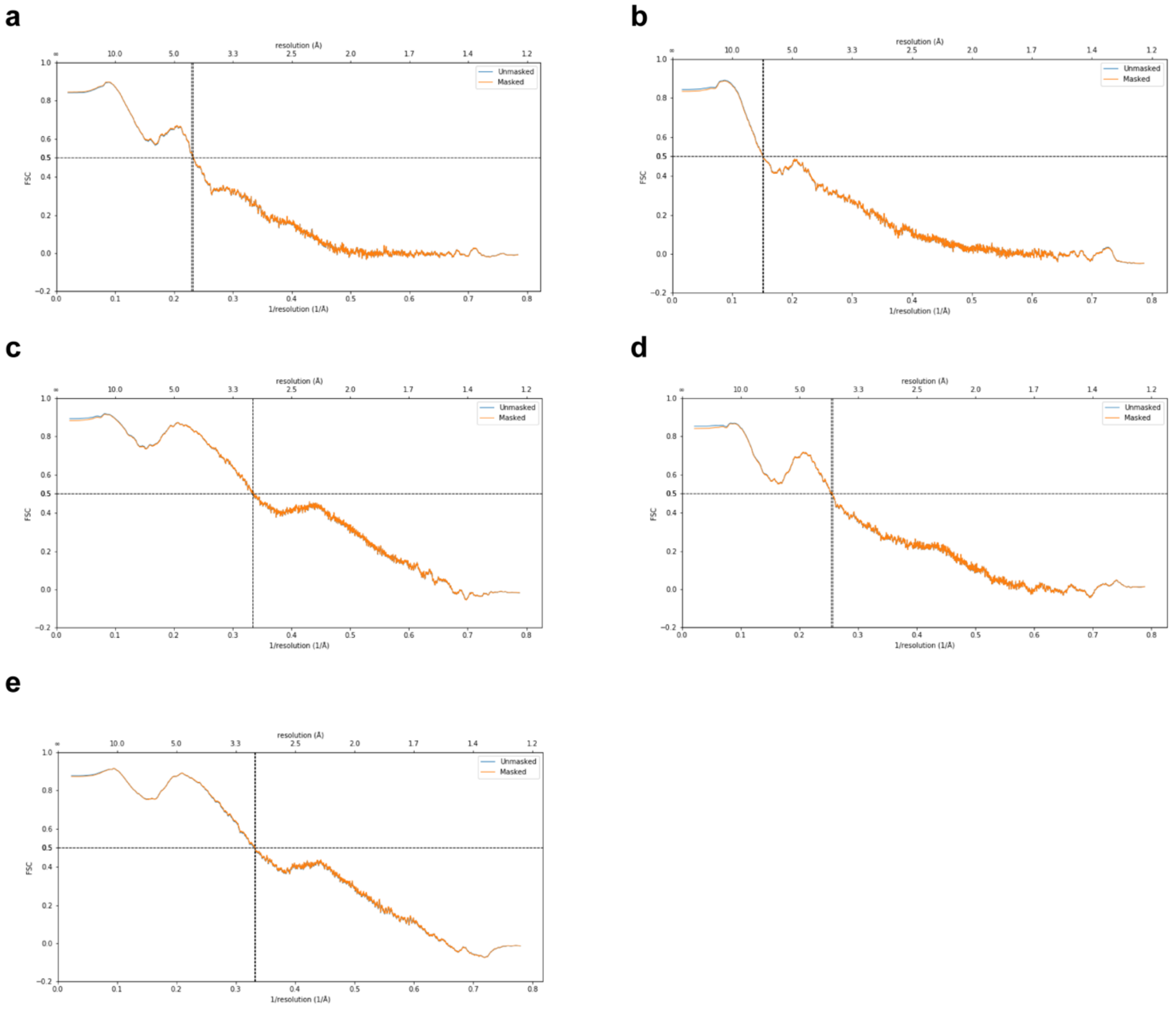

Supplementary Fig. 23. Models-to-map Fourier Shell Correlation (FSC) analysis for the CSN^{5E104A}-SCF dissociation states.

a, FSC curves for dissociation-state-1 CSN-SCF complex. **b**, FSC curves for dissociation-state-2 CSN-SCF complex. **c**, FSC curves for dissociation-state-3 CSN-SCF complex. **d**, FSC curves for dissociation-state-4 CSN-SCF complex. **e**, FSC curves for dissociated CSN^{apo} complex. For each structure, the FSC was calculated between the refined atomic model and the corresponding cryo-EM map with Phenix version 1.21.1 [4]. Both masked (orange) and unmasked (blue) correlations are shown. The resolution at the FSC = 0.5 criterion is indicated by the vertical dashed line. The close agreement between masked and unmasked curves suggests good model-to-map correlation and minimal overfitting.

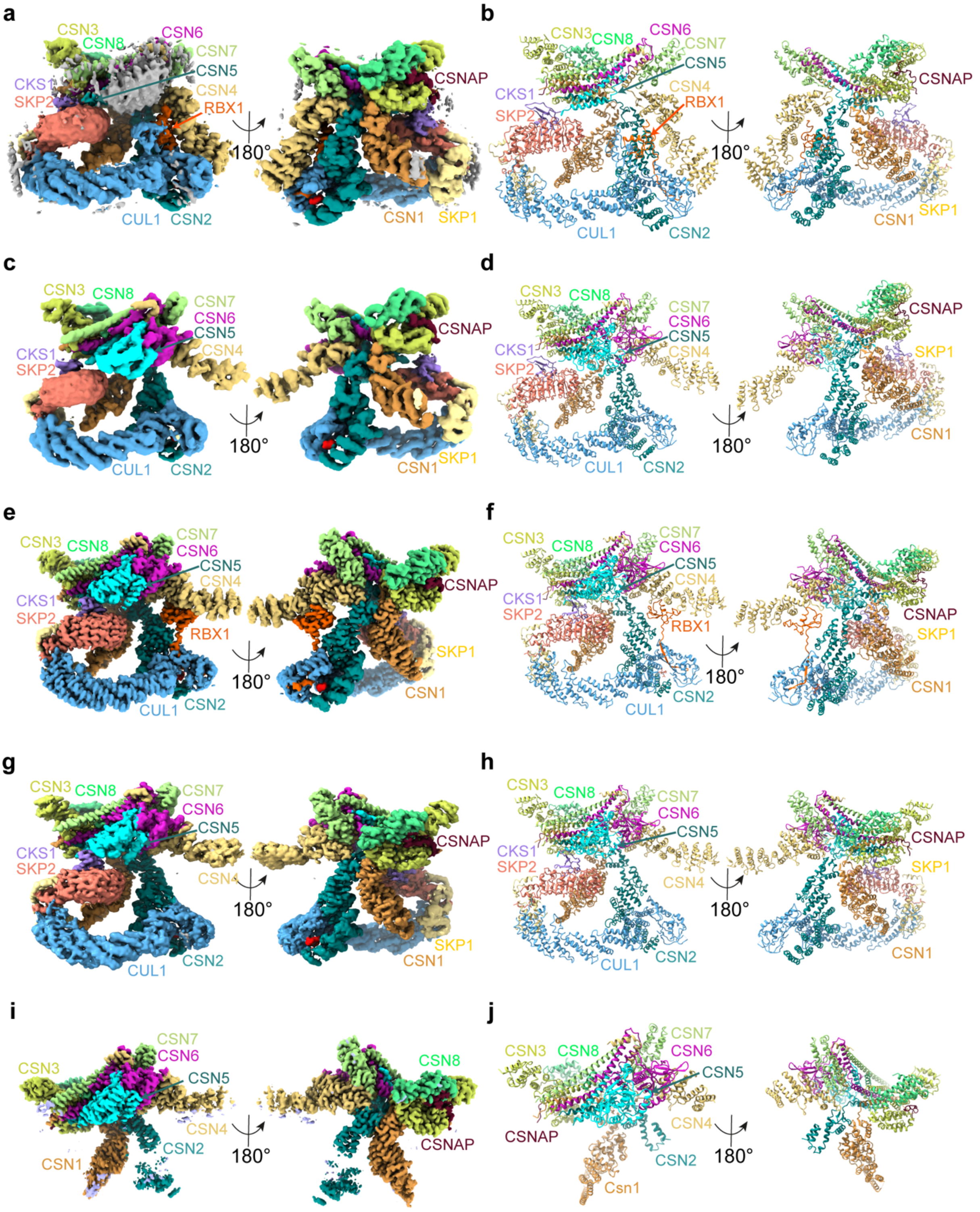

Supplementary Fig. 24. Cryo-EM structures from CSN^{E104A}-SCF dissociation states.

a, Cryo-EM density map of dissociation-state-1. **b,** Molecular model of dissociation-state-1 (ribbon representation). **c,** Cryo-EM density map of dissociation-state-2. **d,** Molecular model of dissociation-state-2 (ribbon representation). **e,** Cryo-EM density

map of dissociation-state-3. **f,** Molecular model of dissociation-state-3 (ribbon representation). **g,** Cryo-EM density map of dissociation-state-4. **h,** Molecular model of dissociation-state-4 (ribbon representation). **i,** Cryo-EM density map of fully dissociated CSN^{apo}. **j,** Molecular model of fully dissociated CSN^{apo} (ribbon representation).

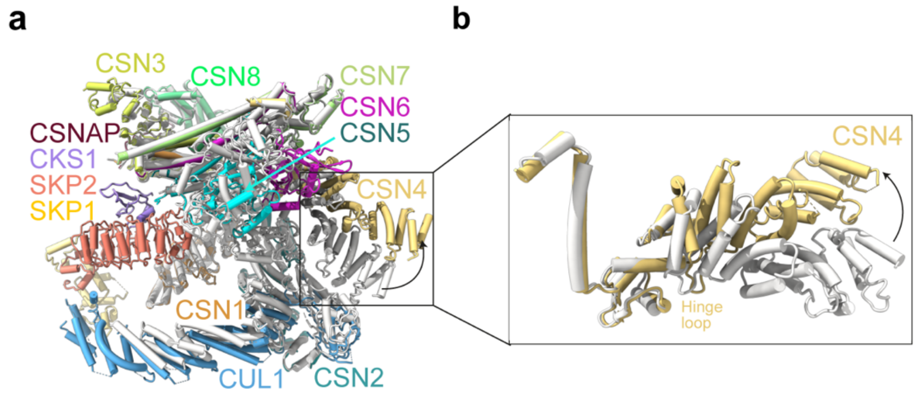

Supplementary Fig. 25. Structural comparison between activated CSN^{5H138A}-^{N8}SCF and CSN^{E104A}-SCF dissociation-state-2.

a, Structural overlay highlighting conformational changes in CSN between activated CSN^{5H138A}-^{N8}SCF (grey) and CSN^{E104A}-SCF dissociation-state-2 (coloured). **b,** Close-up view of the boxed region in (**a**), illustrating the repositioning of the $CSN4^{arm}$ about the $CSN4^{hinge\text{-}loop}$.

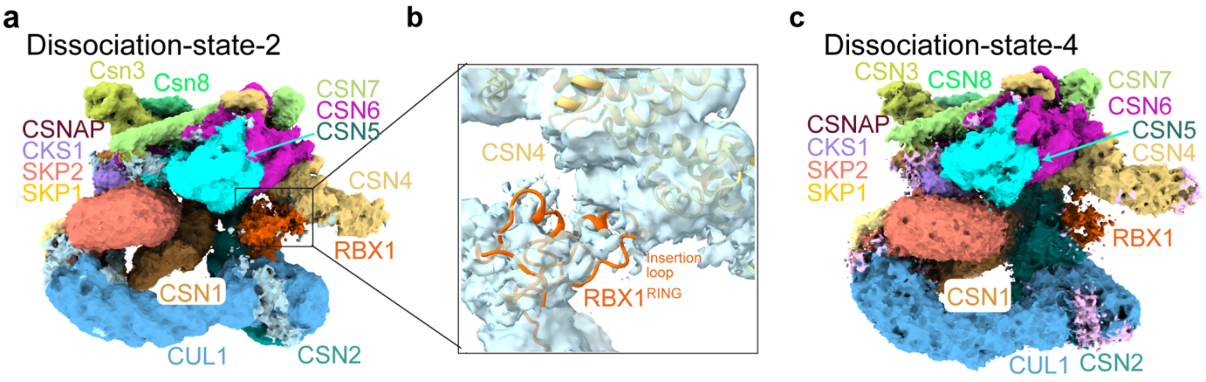

Supplementary Fig. 26. Visualisation of RBX1^{RING} in unprocessed cryo-EM maps of CSN^{E104A}-SCF dissociation-state-2 and dissociation-state-4.

a, Final RELION-4.0 refined cryo-EM map [1] of CSN^{E104A}-SCF dissociation-state-2, revealing weak but distinct density for RBX1^{RING}. **b,** Close-up view of the boxed region in (**a**), featuring a “best-fit” molecular model for RBX1 within the observed density. Notably, a potential contact is evident between the RBX1$^{RING\text{-}Insertion}$ and the CSN4^{arm}. **c,** Final RELION-4.0 refined map of CSN^{E104A}-SCF dissociation-state-4, where only faint density corresponding to a portion of RBX1^{RING} is detectable at very low contour levels.

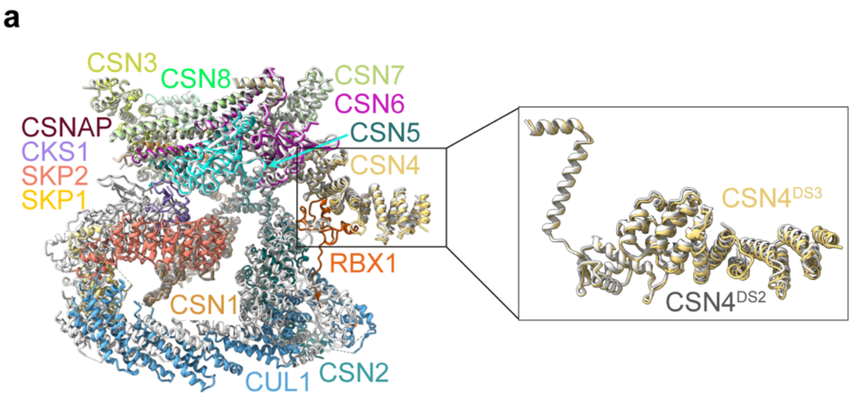

Supplementary Fig. 27. Structural comparison between CSN^{E104A}-SCF dissociation-state-2 and dissociation-state-3.

a, Structural overlay of CSN^{E104A}-SCF dissociation-state-2 (grey) and dissociation-state-3 (coloured). A close-up view of the boxed region reveals that the CSN4^{arm} adopts a similar positioning in both states.

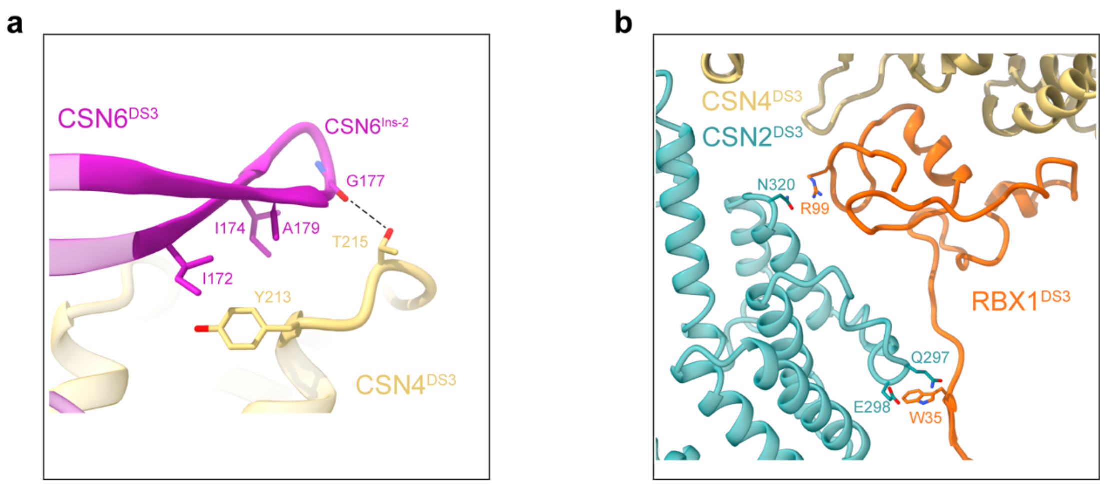

Supplementary Fig. 28. Key interfaces describing CSN^{E104A}-SCF dissociation-state-3.

a, CSN6$^{Ins\text{-}2}$ interacts with CSN4, securing the CSN5-CSN6 MPN domains in an autoinhibited conformation. **b,** The interface between CSN2 and RBX1 in dissociation-state-3 is minimal, indicating a reduced stabilising interaction compared to activated CSN^{5H138A}-^{N8}SCF.

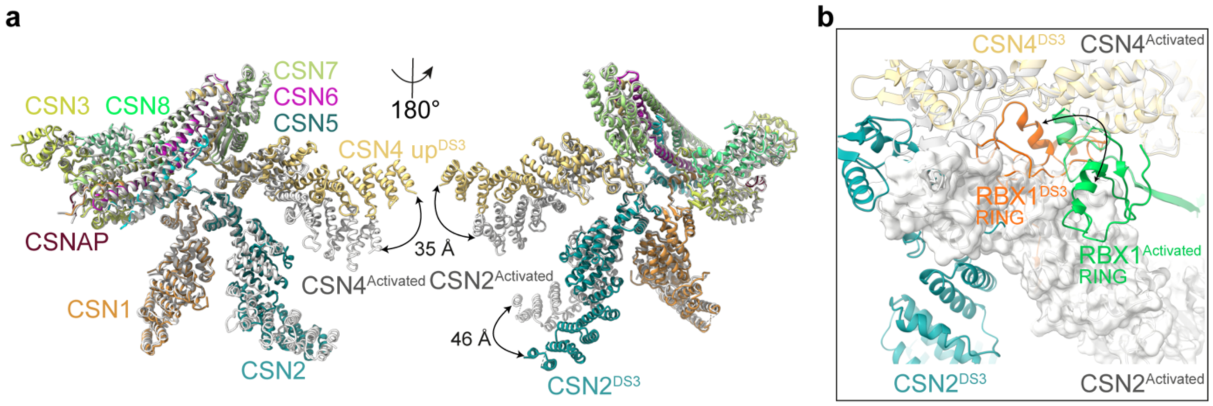

Supplementary Fig. 29. Conformational changes between activated CSN^{5H138A}-^{N8}SCF and CSN^{E104A}-SCF dissociation-state-3.

a, Structural overlay highlighting key conformational changes in CSN2^{arm} and CSN4^{arm} between activated CSN^{5H138A}-^{N8}SCF (grey) and CSN^{E104A}-SCF dissociation-state-3 (coloured). **b,** Remodelling of RBX1^{RING} from its position in activated CSN^{5H138A}-^{N8}SCF (green) to CSN^{E104A}-SCF dissociation-state-3 (orange). In dissociation-state-3, the repositioned RBX1^{RING} results in a significant steric clash with CSN2^{arm} in activated CSN^{5H138A}-^{N8}SCF (grey surface). The structural alignment was performed using pre-activated CSN4 as a reference.

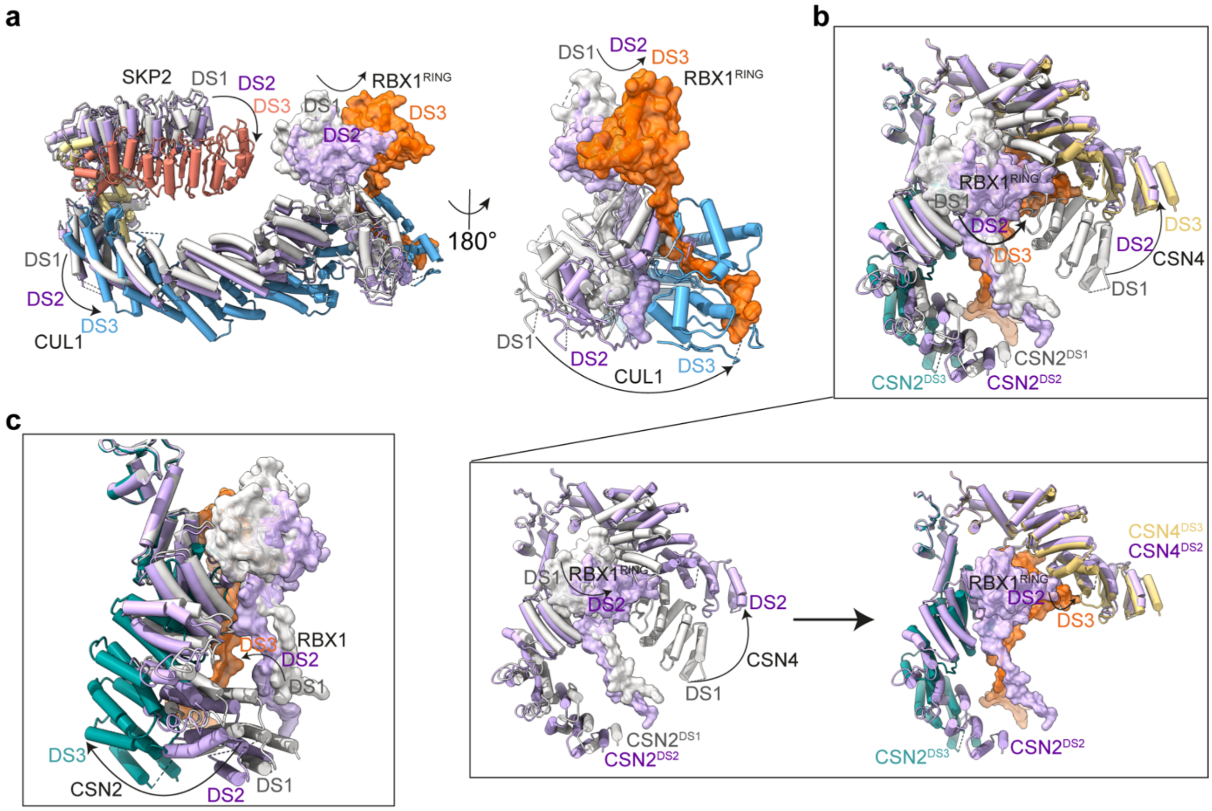

Supplementary Fig. 30. Stepwise conformational changes in SCF, CSN2^{arm} and CSN4^{arm} during CSN^{E104A}-SCF dissociation.

a, Progressive structural transitions within **SCF** across dissociation-state-1 (grey), dissociation-state-2 (purple) and dissociation-state-3 (colour scheme used in previous figures). SCF subunits are depicted in ribbon (tube format) with RBX1 highlighted by an additional semi-transparent surface. The RBX1^{RING} in dissociation-state-2 is a best-fit model for illustrative purposes only. **b,** Stepwise conformational rearrangements of CSN4^{arm} relative to RBX1^{RING}. Top panel: overlay of sequential transitions from dissociation-states-1, -2 and –3. Bottom panel: distinct transitions from dissociation-state-1 (grey) to dissociation-state-2 (purple), followed by dissociation-state-2 (purple) to dissociation-state-3 (colour scheme used in previous figures). **c,** Sequential conformational changes of CSN2^{arm} relative to RBX1, illustrating structural movements across dissociation-states-1, -2 and -3.

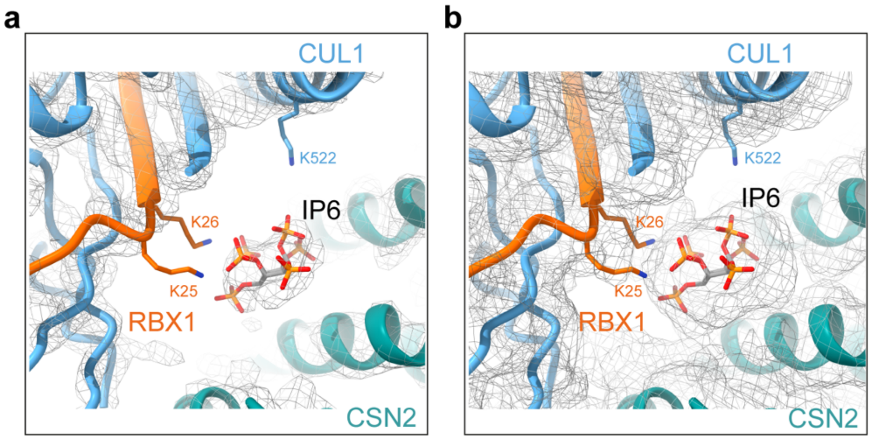

Supplementary Fig. 31. CSN2-bound IP6 and its interactions with CUL1/RBX1 in CSN^{E104A}-SCF dissociation-state-3.

a, Cryo-EM density map from CSN^{E104A}-SCF dissociation-state-3, highlighting the density for IP6 bound to CSN2. **b,** Low-contour view of the same map in (**a**), revealing side chain densities for RBX1^{K25}, RBX1^{K26} and CUL1^{K522}, suggesting potential interaction sites between IP6 and CUL1/RBX1 in dissociation-state-3.

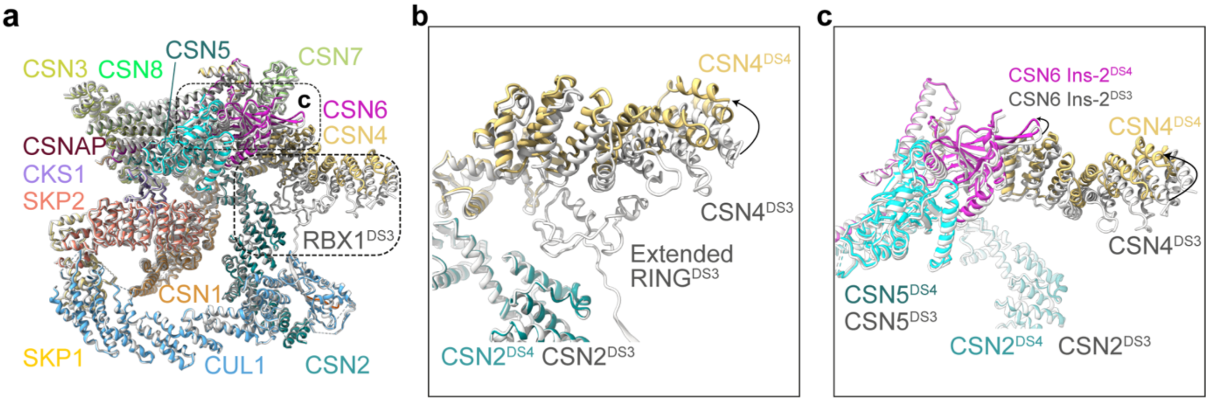

Supplementary Fig. 32. Structural comparison between CSN^{E104A}-SCF dissociation-state-3 and dissociation-state-4.

a, Structural overlay highlighting key conformational changes in CSN between CSN^{E104A}-SCF dissociation-state-3 (grey) and CSN^{E104A}-SCF dissociation-state-4 (coloured). **b,** Close-up view of the boxed region in (**a**), illustrating an upward shift of the CSN4^{arm} in dissociation-state-4, which disrupts the RBX1^{RING} interface observed in dissociation-state-3. **c,** Close-up view of the boxed region in (**a**), showing the repositioning of CSN6$^{Ins\text{-}2}$ in dissociation-state-4, which maintains its interface with CSN4^{arm}.

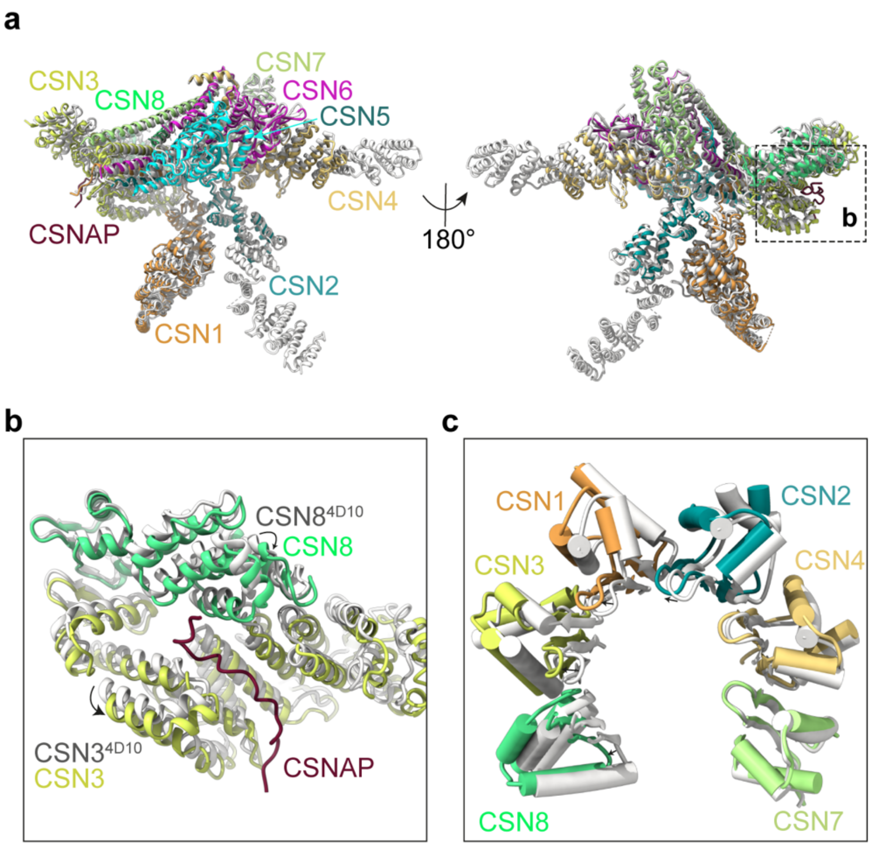

Supplementary Fig. 33. Structural rearrangements in CSN upon CSNAP incorporation.

a, Structural overlay of the 9-subunit CSN complex (coloured) and the 8-subunit CSN complex (PDB: 4D10) (grey). **b,** Close-up view of the boxed region in (**a**), illustrating structural adjustments in CSN3 and CSN8 upon CSNAP incorporation. **c,** Conformational changes within the PCI ring upon incorporation of CSNAP.

Supplementary Tables

Supplementary Table 1 Cryo-EM data collection and refinement statistics of CSN^{5H138A}-^{N8}SCF

	CSN^{5H138A}-^{N8}SCF	
	Pre-activated<br>(EMD-53252)<br>(PDB 9QO0)	Activated<br>(EMD-53253)<br>(PDB 9QO1)
**Data collection and processing**		
Magnification	96,000	96,000
Voltage (kV)	300	300
Electron exposure (e–/Å^2)	47	47
Defocus range (μm)	-0.5 to -5	-0.5 to -5
Pixel size (Å)	1.08	1.08
Symmetry imposed	C1	C1
Initial particle images (no.)	3,570,389	3,570,389
Final particle images (no.)	400,018	556,830
Map resolution (Å)	3.3	3.2
FSC threshold	0.143	0.143
Map resolution range (Å)	3.0–7.3	3.0–9.2
**Refinement**		
Initial model used (PDB code)	6R7I, 1LDK, 2ASS	
Model resolution (Å)	3.3	3.2
FSC threshold	0.143	0.143
Model resolution range (Å)	3.0–7.3	3.0–9.2
Map sharpening *B* factor (Å^2)	-73.34879	-71.93767
**Model composition**		
Non-hydrogen atoms	29, 372	25, 396
Protein residues	3744	3165
Ligands	IHP: 1; ZN: 3	IHP: 1; ZN: 3
B factors (Å^2)		
Protein	86.52	80.3
Ligand	119.98	132.88
**R.m.s. deviations**		

Bond lengths (Å)	0.003	0.003
Bond angles (°)	0.576	0.587
**Validation**		
MolProbity score	1.95	1.81
Clashscore	4.98	3.51
Poor rotamers (%)	3.24	2.93
Ramachandran plot		
Favored (%)	95.66	95.53
Allowed (%)	4.34	4.47
Disallowed (%)	0.00	0.00

Supplementary Table 2 Cryo-EM data collection and refinement statistics of CSN^{5E104A}-SCF

	CSN^{5E104A}-SCF				CSN^{5E104A}
	Dissociation-state-1 (EMD-53254) (PDB 9QO2)	Dissociation-state-2 (EMD-53255) (PDB 9QO3)	Dissociation-state-3 (EMD-53256) (PDB 9QO4)	Dissociation-state-4 (EMD-53257) (PDB 9QO5)	CSN^{Apo} (EMD-53258) (PDB 9QO6)
**Data collection and processing**					
Magnification	96,000	96,000	96,000	96,000	96,000
Voltage (kV)	300	300	300	300	300
Electron exposure (e–/Å^2)	47	47	47	47	47
Defocus range (μm)	-0.5 to -5	-0.5 to -5	-0.5 to -5	-0.5 to -5	-0.5 to -5
Pixel size (Å)	1.08	1.08	1.08	1.08	1.08
Symmetry imposed	C1	C1	C1	C1	C1
Initial particle images (no.)	3,830,233	3,830,233	3,830,233	3,830,233	3,830,233
Final particle images (no.)	1,015,152	54,098	409,455	131,512	582,407
Map resolution (Å)	3.8	4.6	3.0	4.0	3.0
FSC threshold	0.143	0.143	0.143	0.143	0.143
Map resolution range (Å)	3.4–10.3	3.9–10.8	2.6–5.2	3.3–8.9	2.8–7.8
**Refinement**					
Initial model used (PDB code)					
Model resolution (Å)	3.8	4.6	3.0	4.0	3.0
FSC threshold	0.143	0.143	0.143	0.143	0.143
Model resolution range (Å)	3.4–10.3	3.9–10.8	2.6–5.2	3.3–8.9	2.8–7.8
Map sharpening *B* factor (Å^2)	-47.22885	-16.67099	-30.74515	-19.74951	-34.71830
**Model composition**					

Non-hydrogen atoms	26, 526	28,849	30, 680	29,818	17385
Protein residues	3363	3659	3807	3700	2175
Ligands	IHP: 1; ZN: 3	IHP: 1; ZN:1	IHP: 1; ZN: 4	IHP: 1; ZN: 1	ZN: 1
B factors ($Å^2$)					
Protein	140.02	196.76	77.34	105.26	70.9
Ligand	131.90	214.51	121.39	126.2	27.64
**R.m.s. deviations**					
Bond lengths (Å)	0.004	0.003	0.005	0.005	0.005
Bond angles (°)	0.776	0.653	1.054	1.052	1.018
**Validation**					
MolProbity score	2.06	1.58	1.78	1.83	1.54
Clashscore	6.1	4.79	3.86	5.13	2.64
Poor rotamers (%)	2.79	0.06	2.22	2.25	1.63
Ramachandran plot					
Favored (%)	94.35	95.18	95.06	95.82	95.3
Allowed (%)	5.65	4.79	4.94	4.16	4.70
Disallowed (%)	0.00	0.03	0.00	0.03	0.00

Supplementary Table 3 Oligonucleotide sequences

Name	Sequence
CUL1_Q728A_K726A_F	gcaCACgcGCAGTTACTTGGCGAGGTCCT
CUL1_Q728A_K726A_R	GTAACTGcgcGTGtgcCAGAACCTTCCTCATCTTCATGATTCTCAC
CSN5_E122K_1_F	cgtgtgcagttgtctggacctcaggAGGCAGAGAAATACGTTCTGC
CSN5_E122K_1_R	gtttcgcattttttatgtatgcagccatg
CSN5_E122K_2_F	atacataaaaaatgcgaaacaggttggcc
CSN5_E122K_2_R	aatttaccgaacaactccgcggccgggaagccgatctcgg
CSN5_M117R_1_R	tgtatgcagcCCGGTATTCATATGCAGCAGCCTG
CSN5_M117R_2_F	tgaataccggGCTGCATACATAgAAAATGCGAAACAGG
CSN5_L157G/F161G_1_R	atggttcctgGCCCTGCTGATTGCCCATCtgagtaCTAACATCAA
CSN5_L157G/F161G_2_F	tcagcagggcCAGGAACCATTTGTAGCAGTGGTG
CSN5_T105F_1_R	catttactcgGAATTCAGTGCCCTCCAC
CSN5_T105F_2_F	cactgaattcCGAGTAAATGCTCAGGCTGCTG
CUL1_R741E_F	GCTGTCCTCCgagTTCAAACCTCGAGTC
CUL1_R741E_R	TGAGTGAGGACCTCGCCA
CUL1_R717E_F	GGCCATCGTGgaaATCATGAAGATGAGG
CUL1_R717E_R	GCCTGAATCAGTAGTTTGCGGTCTTC

Uncropped SDS-PAGE gels in Supplementary Figures

Supplementary Fig.1a

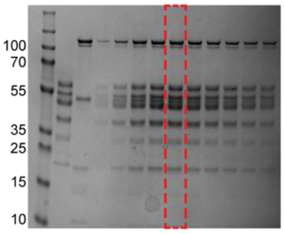

Supplementary Fig.11a

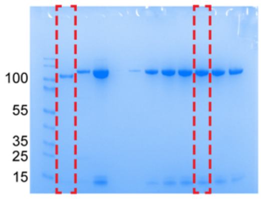

Supplementary Fig.11b

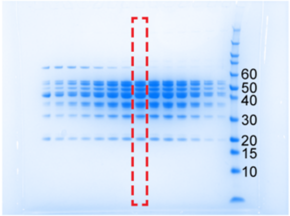

Supplementary Fig.11c

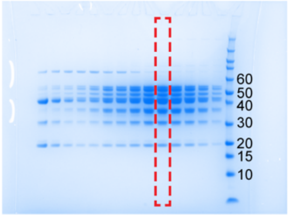

Supplementary Fig.11d

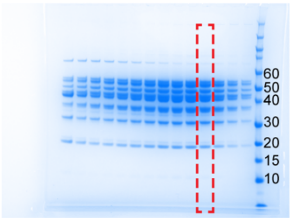

Supplementary Fig.11e

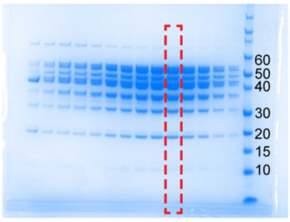

Supplementary Fig.11f

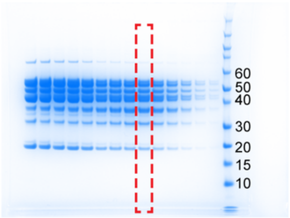

Supplementary Fig.11g

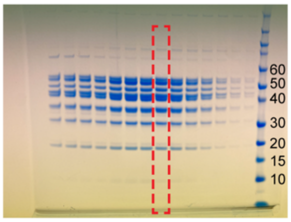

Supplementary Fig.11h

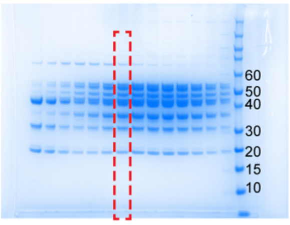

Supplementary Fig.11i

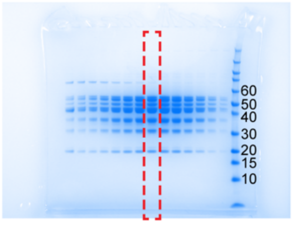

Supplementary Fig.11j

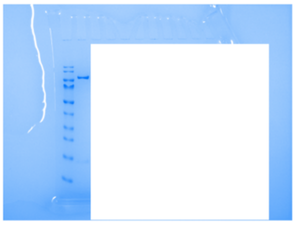

Supplementary Fig.11j

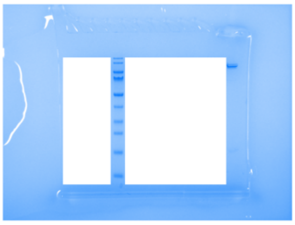

Supplementary Fig.11k

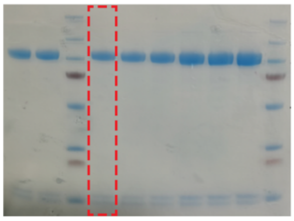

Supplementary Fig.11k

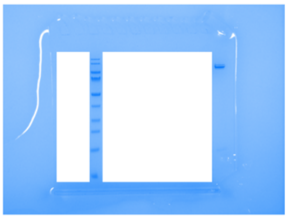

Supplementary Fig.22a-1

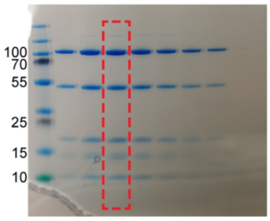

Supplementary Fig.22a-2

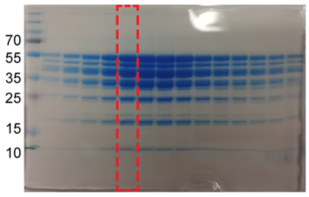

Supplementary Reference

1 Kimanius, D., Dong, L., Sharov, G., Nakane, T. & Scheres, S. H. W. New tools for automated cryo-EM single-particle analysis in RELION-4.0. *Biochem J* **478**, 4169-4185 (2021). https://doi.org:10.1042/BCJ20210708

2 Zhong, E. D., Bepler, T., Berger, B. & Davis, J. H. CryoDRGN: reconstruction of heterogeneous cryo-EM structures using neural networks. *Nat Methods* **18**, 176-185 (2021). https://doi.org:10.1038/s41592-020-01049-4

3 Tan, Y. Z. *et al.* Addressing preferred specimen orientation in single-particle cryo-EM through tilting. *Nat Methods* **14**, 793-796 (2017). https://doi.org:10.1038/nmeth.4347

4 Adams, P. D. *et al.* PHENIX: a comprehensive Python-based system for macromolecular structure solution. *Acta Crystallogr D Biol Crystallogr* **66**, 213-221 (2010). https://doi.org:10.1107/S0907444909052925

5 Pintilie, G. *et al.* Measurement of atom resolvability in cryo-EM maps with Q-scores. *Nat Methods* **17**, 328-334 (2020). https://doi.org:10.1038/s41592-020-0731-1

6 Edgar, R. C. MUSCLE: multiple sequence alignment with high accuracy and high throughput. *Nucleic Acids Res* **32**, 1792-1797 (2004). https://doi.org:10.1093/nar/gkh340

7 Angers, S. *et al.* Molecular architecture and assembly of the DDB1-CUL4A ubiquitin ligase machinery. *Nature* **443**, 590-593 (2006). https://doi.org:10.1038/nature05175

8 Fischer, E. S. *et al.* The molecular basis of CRL4DDB2/CSA ubiquitin ligase architecture, targeting, and activation. *Cell* **147**, 1024-1039 (2011). https://doi.org:10.1016/j.cell.2011.10.035

9 Duda, D. M. *et al.* Structural insights into NEDD8 activation of cullin-RING ligases: conformational control of conjugation. *Cell* **134**, 995-1006 (2008). https://doi.org:10.1016/j.cell.2008.07.022

10 Hopf, L. V. M. *et al.* Structure of CRL7(FBXW8) reveals coupling with CUL1-RBX1/ROC1 for multi-cullin-RING E3-catalyzed ubiquitin ligation. *Nat Struct Mol Biol* **29**, 854-862 (2022). https://doi.org:10.1038/s41594-022-00815-6

11 Horn-Ghetko, D. *et al.* Noncanonical assembly, neddylation and chimeric cullin-RING/RBR ubiquitylation by the 1.8 MDa CUL9 E3 ligase complex. *Nat Struct Mol Biol* **31**, 1083-1094 (2024). https://doi.org:10.1038/s41594-024-01257-y
