# Supplementary material for: Structural basis of CSN-mediated SCF deneddylation
Source: Nat Commun. 2026 Jan 23;17:951. doi: 10.1038/s41467-025-67566-y (PMC12848000; doi:10.1038/s41467-025-67566-y)
Supplement: Supplementary file 2 — Description of Additional Supplementary File [file 41467_2025_67566_MOESM2_ESM.pdf]

The Description of Additional Supplementary Files

Supplementary Movie 1: Morphing between the autoinhibited, pre-activated and activated states of the CSN complex. The movie begins with a molecular model of CSN in the autoinhibited CSN^{apo} state (PDB: 4D10) [1], shown together with the NEDD8–CUL1^{WHB} module from the active UBE2D2-^{N8}SCF complex (PDB: 6TTU) [2]. The morph then transitions to the pre-activated CSN^{5H138A}-^{N8}SCF complex. Upon engagement with a neddylated SCF complex, the N-terminal helical arms of CSN2 and CSN4 converge, clamping around the CUL1^{CTD} and RBX1^{RING} domains. This conformational shift is accompanied by the disruption of the CSN4-CSN6 interface, which permits structural remodelling of the CSN5-CSN6 MPN domain module. As a result, the CSN5 active site is repositioned closer to NEDD8. The final transition shows the activated CSN^{5H138A}-^{N8}SCF complex, in which further targeted rearrangements around CSN5, CUL1^{WHB} and NEDD8 generate interaction surfaces that are specific to the activated state. The C-terminal tail of NEDD8 is precisely oriented within the CSN5 active site.

Supplementary Movie 2: Morphing between the autoinhibited, pre-activated and activated states of CSN5 within the CSN complex. The movie begins with CSN5 in the autoinhibited CSN^{apo} state (PDB: 4D10) [1], shown together with the NEDD8–CUL1^{WHB} module from the active UBE2D2-^{N8}SCF complex (PDB: 6TTU) [2]. In this state, the catalytic zinc-binding site of CSN5 is blocked by CSN5^{E104} from the CSN5^{Ins-1} loop, which acts as a fourth ligand to the zinc ion, keeping the isopeptidase inactive. The morph then transitions to the pre-activated CSN^{5H138A}-^{N8}SCF complex, in which the CSN5^{Ins-1} loop is displaced from the active site, relieving autoinhibition and enabling access for the incoming isopeptide bond. Finally, the movie proceeds to the activated CSN^{5H138A}-^{N8}SCF state of the complex, where further conformational remodelling of CSN5, CUL1^{WHB} and NEDD8 aligns the isopeptide bond precisely within the active site, positioning it for catalysis. The sequence concludes with a zoomed-in view of the zinc-coordination site and substrate-binding geometry within the CSN5 catalytic pocket.

Supplementary Movie 3: Morphing between CSN^{E104A}-SCF dissociation states (1-4), illustrating the sequential mechanism of CSN-SCF disassembly. The movie begins with a molecular model of dissociation-state-1, which most closely resembles the activated CSN^{5H138A}-^{N8}SCF complex. In the absence of NEDD8 however, the CUL1^{WHB} exhibits increased flexibility. Morphing to dissociation-state-2, the CSN4^{arm} disengages from CUL1^{CTD} and adopts an "up" conformation. The RBX1^{RING} domain tracks this motion, while CSN5 and CSN6 retract towards their CSN^{apo} positions. In dissociation-state-3, the RBX1^{RING} extends further, establishing an interface with the disengaged CSN4^{arm}. The CSN2^{arm} shifts laterally away from the CSN core, accompanied by a displacement of the CSN2-bound CUL1^{CTD}. This repositioning drives a reorientation of the SR within the CSN complex. Finally, the architecture of dissociation-state-4 is broadly similar to state-3; however, the CSN4^{arm} adopts a "fully up" conformation, disrupting its interface with the RBX1^{RING}. The sequence concludes with the CSN^{apo} state and the inactive SCF complex (a superimposed complex with CUL1-RBX1-SKP1 from PDB: 1LDK [3] and SKP1/SKP2/CKS1 from PDB: 2ASS [4]).

Supplementary Movie 4: Morphing between structural snapshots that capture distinct mechanistic stages on the CSN-mediated deneddylation cycle of SCF. The sequence begins with a molecular model of the pre-activated CSN^{5H138A}-^{N8}SCF complex, progresses to the activated CSN^{5H138A}-^{N8}SCF state, and

concludes with the stepwise dissociation pathway of CSN^{E104A}-SCF, depicted through dissociation states 1-4.
