# Supplementary material for: Structural basis of CSN-mediated SCF deneddylation
Source: Nat Commun. 2026 Jan 23;17:951. doi: 10.1038/s41467-025-67566-y (PMC12848000; doi:10.1038/s41467-025-67566-y)
Supplement: Supplementary file 7 — Reporting Summary [file 41467_2025_67566_MOESM7_ESM.pdf]

Corresponding author(s): Radoslav I. Enchev

Last updated by author(s): Oct 2, 2025

Reporting Summary

Nature Portfolio wishes to improve the reproducibility of the work that we publish. This form provides structure for consistency and transparency in reporting. For further information on Nature Portfolio policies, see our Editorial Policies and the Editorial Policy Checklist.

Statistics

For all statistical analyses, confirm that the following items are present in the figure legend, table legend, main text, or Methods section.

n/a	Confirmed	
☐	☒	The exact sample size (*n*) for each experimental group/condition, given as a discrete number and unit of measurement
☐	☒	A statement on whether measurements were taken from distinct samples or whether the same sample was measured repeatedly
☒	☐	The statistical test(s) used AND whether they are one- or two-sided<br>*Only common tests should be described solely by name; describe more complex techniques in the Methods section.*
☒	☐	A description of all covariates tested
☒	☐	A description of any assumptions or corrections, such as tests of normality and adjustment for multiple comparisons
☐	☒	A full description of the statistical parameters including central tendency (e.g. means) or other basic estimates (e.g. regression coefficient) AND variation (e.g. standard deviation) or associated estimates of uncertainty (e.g. confidence intervals)
☒	☐	For null hypothesis testing, the test statistic (e.g. *F*, *t*, *r*) with confidence intervals, effect sizes, degrees of freedom and *P* value noted<br>*Give P values as exact values whenever suitable.*
☒	☐	For Bayesian analysis, information on the choice of priors and Markov chain Monte Carlo settings
☒	☐	For hierarchical and complex designs, identification of the appropriate level for tests and full reporting of outcomes
☒	☐	Estimates of effect sizes (e.g. Cohen's *d*, Pearson's *r*), indicating how they were calculated

Our web collection on statistics for biologists contains articles on many of the points above.

Software and code

Policy information about availability of computer code

Data collection	UNICORN 7.6 (version Build 7.6.0.1306)<br>EPU software (Thermo Fisher Scientific)<br>Vitrobot Mark IV FEI (serve: https://www.thermofisher.com/us/en/home/electron-microscopy/products/sample-preparation-equipment-em/vitrobot/instruments/vitrobot-mark-iv.html)<br>Typhoon FLA 9500 (Cytiva)<br>Biacore S200 instrument (Cytiva)<br>ChemiDoc XRS+ System (BIO-RAD)
Data analysis	RELION-4.0<br>SPHIRE-crYOLO<br>cryoSPARC v3.3.2<br>CryoDRGN v1.0.0<br>DeepEMhancer<br>Remote 3DFSC Processing Server (https://3dfsc.salk.edu/upload/)<br>UCSF Chimera v1.16<br>UCSF ChimeraX v1.5<br>Coot v0.9.6<br>Namdinator (https://namdinator.au.dk/)<br>Phenix v1.21.1<br>MolProbity server (http://molprobity.biochem.duke.edu/)<br>wwPDB Validation (https://deposit-pdbe.wwpdb.org/)

GraphPad Prism Version 10.0
Biacore S200 Evaluation Software v1.0 (Cytiva)

For manuscripts utilizing custom algorithms or software that are central to the research but not yet described in published literature, software must be made available to editors and reviewers. We strongly encourage code deposition in a community repository (e.g. GitHub). See the Nature Portfolio guidelines for submitting code & software for further information.

Data

Policy information about availability of data

All manuscripts must include a data availability statement. This statement should provide the following information, where applicable:

- Accession codes, unique identifiers, or web links for publicly available datasets
- A description of any restrictions on data availability
- For clinical datasets or third party data, please ensure that the statement adheres to our policy

Coordinates and maps associated with data reported in this manuscript were deposited to the Electron Microscopy Data Bank (EMDB) and Protein Data Bank (PDB) with accession numbers EMD-53252 and PDB 9QO0 (pre-activated CSN5H138A-N8SCF), EMD-53253 and PDB 9QO1 (activated CSN5H138A-N8SCF), EMD-53254 and PDB 9QO2 (CSNE104A-SCF dissociation-state-1), EMD-53255 and PDB 9QO3 (CSNE104A-SCF dissociation-state-2), EMD-53256 and PDB 9QO4 (CSNE104A-SCF dissociation-state-3), EMD-53257 and PDB 9QO5 (CSNE104A-SCF dissociation-state-4), EMD-53258 and PDB 9QO6 (9-subunit CSNApo).

Research involving human participants, their data, or biological material

Policy information about studies with human participants or human data. See also policy information about sex, gender (identity/presentation), and sexual orientation and race, ethnicity and racism.

Reporting on sex and gender	N/A, no human subjects data
Reporting on race, ethnicity, or other socially relevant groupings	N/A, no human subjects data
Population characteristics	N/A, no human subjects data
Recruitment	N/A, no human subjects data
Ethics oversight	N/A, no human subjects data

Note that full information on the approval of the study protocol must also be provided in the manuscript.

Field-specific reporting

Please select the one below that is the best fit for your research. If you are not sure, read the appropriate sections before making your selection.

☒ Life sciences ☐ Behavioural & social sciences ☐ Ecological, evolutionary & environmental sciences

For a reference copy of the document with all sections, see nature.com/documents/nr-reporting-summary-flat.pdf

Life sciences study design

All studies must disclose on these points even when the disclosure is negative.

Sample size	No statistical methods were used to predetermine sample size. Sample sizes for all experiments were chosen based on commonly accepted standards in the field and prior experience with similar experimental systems. For SPR and deneddylation assay, data reproducibility across independent biological replicates (typically n = 3) was used to assess robustness. For cryo-EM structural analysis, the number of particles included in the final reconstruction was determined by data quality and homogeneity during processing, not by a priori statistical calculation. The chosen sample sizes were sufficient to ensure consistent and reproducible results across experiments.
Data exclusions	No data were excluded.
Replication	The protein purifications were repeated at least three times with similar results. All key experiments were independently repeated at least three times. Representative datasets are presented in the figures.
Randomization	Random grouping was not used, because the experiments (like cryo-EM, SPR, deneddylation assay) were done with purified proteins, not with living samples or biological replicates.
Blinding	No blinding was used. All biochemical experiments involved comparing the binding affinities of wild-type and mutant proteins under in vitro conditions. Blinding is not standard practice for these types of assays in the field.

Reporting for specific materials, systems and methods

We require information from authors about some types of materials, experimental systems and methods used in many studies. Here, indicate whether each material, system or method listed is relevant to your study. If you are not sure if a list item applies to your research, read the appropriate section before selecting a response.

Materials & experimental systems

n/a	Involved in the study
☒	☐ Antibodies
☐	☒ Eukaryotic cell lines
☒	☐ Palaeontology and archaeology
☒	☐ Animals and other organisms
☒	☐ Clinical data
☒	☐ Dual use research of concern
☒	☐ Plants

Methods

n/a	Involved in the study
☒	☐ ChIP-seq
☒	☐ Flow cytometry
☒	☐ MRI-based neuroimaging

Eukaryotic cell lines

Policy information about cell lines and Sex and Gender in Research

Cell line source(s)	Sf9 (Spodoptera frugiperda; ATCC CRL 1711)<br>High Five/BTI Tn 5B1 4 (Trichoplusia ni; Thermo Fisher Scientific, Cat. no. B855 02)
Authentication	Insect cell lines were authenticated by the suppliers; identity further confirmed in-house by morphology, growth rate and baculovirus production characteristics. STR profiling is not applicable for insect lines.
Mycoplasma contamination	Not tested; no morphological signs of contamination were observed during routine culture of Sf9 and High Five insect cells
Commonly misidentified lines<br>(See ICLAC register)	Neither Sf9 nor High Five are listed in the ICLAC database of misidentified cell lines

Plants

Seed stocks	N/A
Novel plant genotypes	N/A
Authentication	N/A
